# Interneuron Heterotopia in the Lis1 Mutant Mouse Cortex Underlies a Structural and Functional Schizophrenia-Like Phenotype

**DOI:** 10.3389/fcell.2021.693919

**Published:** 2021-07-13

**Authors:** Raquel Garcia-Lopez, Ana Pombero, Alicia Estirado, Emilio Geijo-Barrientos, Salvador Martinez

**Affiliations:** ^1^Instituto de Neurociencias, UMH-CSIC, Alicante, Spain; ^2^Centro de Investigación Biomédica En Red en Salud Mental-CIBERSAM-ISCIII, Valencia, Spain

**Keywords:** *LIS1* gene, schizophrenia, GABAergic system, interneurons, hippocampus, mPFC, c-fos

## Abstract

*LIS1* is one of the principal genes related to Type I lissencephaly, a severe human brain malformation characterized by an abnormal neuronal migration in the cortex during embryonic development. This is clinically associated with epilepsy and cerebral palsy in severe cases, as well as a predisposition to developing mental disorders, in cases with a mild phenotype. Although genetic variations in the *LIS1* gene have been associated with the development of schizophrenia, little is known about the underlying neurobiological mechanisms. We have studied how the *Lis1* gene might cause deficits associated with the pathophysiology of schizophrenia using the *Lis1/sLis1* murine model, which involves the deletion of the first coding exon of the *Lis1* gene. Homozygous mice are not viable, but heterozygous animals present abnormal neuronal morphology, cortical dysplasia, and enhanced cortical excitability. We have observed reduced number of cells expressing GABA-synthesizing enzyme glutamic acid decarboxylase 67 (GAD67) in the hippocampus and the anterior cingulate area, as well as fewer parvalbumin-expressing cells in the anterior cingulate cortex in *Lis1/sLis1* mutants compared to control mice. The cFOS protein expression (indicative of neuronal activity) in *Lis1/sLis1* mice was higher in the medial prefrontal (mPFC), perirhinal (PERI), entorhinal (ENT), ectorhinal (ECT) cortices, and hippocampus compared to control mice. Our results suggest that deleting the first coding exon of the *Lis1* gene might cause cortical anomalies associated with the pathophysiology of schizophrenia.

## Introduction

Abnormal neuronal migration during brain development results in several brain organization alterations. One of the most significant cortical abnormalities is lissencephaly (Reiner et al., 1995; Kato and Dobyns, 2003; Barkovich et al., 2005; Wynshaw-Boris, 2007). This developmental alteration produces severe symptoms that include seizures, intellectual retardation, and a higher risk of developing psychotic disorders like schizophrenia (Dobyns et al., 1993; Tabarés-Seisdedos et al., 2008). Schizophrenia (SZ) is a chronic and serious psychiatric illness that occurs in 1% of the global population (Freedman, 2003) and which is caused by both genetic and environmental factors. The symptoms of SZ fall into three categories: (1) positive symptoms that include hallucinations, delusions, cognitive deficits, and movement disorders; (2) negative symptoms, such as the disruption of normal emotions and behaviors; and (3) cognitive symptoms: attention deficit and memory problems (Ross et al., 2006; Insel, 2010).

The precise cause of SZ remains unclear, postmortem studies have shown a decrease of GABA-synthesizing enzyme (GAD67) together with a reduction in the number of parvalbumin (PV)+ interneurons in the medial prefrontal cortex (mPFC) (Hashimoto et al., 2003, 2008; Reynolds et al., 2004; Woo et al., 2008; Glausier et al., 2014; Hoftman et al., 2015). Other GABAergic subtypes of interneurons, such as cholecystokinin, vasoactive intestinal peptide, somatostatin, neuropeptide Y, and calbindin (CB) have also been reported to be less expressed in the mPFC of SZ patients (Hashimoto et al., 2003, 2008; Fung et al., 2010, 2014). These deficits in the GABAergic interneurons seem to be related to the alteration of PFC-dependent cognitive functions in SZ patients. Indeed, PFC is implicated in important cognitive processes such as attention, memory, and behavioral changes (Heidbreder and Groenewegen, 2003; Gabbott et al., 2005; Briand et al., 2007).

Furthermore, functional failures of N-methyl-D-aspartate (NMDA) receptors, typically found in GABAergic neurons, affect the maturation of GABAergic interneurons (Thomases et al., 2013). Interestingly, previous studies provided evidence that NMDA receptor antagonists, such as ketamine, MK-801, and phencyclidine (PCP), reproduce schizophrenia-like symptoms in mice. For this reason, blocking NMDA receptors is the approach most widely used to mimic SZ symptoms in animal models (Jentsch and Roth, 1999; Ozdemir et al., 2012; Gilabert-Juan et al., 2013; Kim et al., 2014; Li J.-T. et al., 2016; Castillo-Gómez et al., 2017). In mice, PCP treatments generate abnormal behaviors (Boyce et al., 1991), including hyperactivity, stereotypic behaviors, and motor dysfunction (Castellani and Adams, 1981; Nabeshima et al., 1987). Accordingly, PCP produces schizophrenia-like psychosis, and increases psychotic symptoms in SZ patients (Javitt and Zukin, 1991). Moreover, several studies have reported that PCP induces cFOS protein expression in pontine nuclei and the thalamus, as well as in cortical regions including the retrosplenial, pyriform, cingulate, and frontal cortices. These results suggest a potential relationship between increased cFOS and the psychotic effects of the NMDA receptor antagonists (Abdel-Naby Sayed et al., 2001).

The LIS1 complex is a tetramer intracellular enzyme composed of two catalytic subunits, PAFAH-1B3 (ALPHA1) and PAFAH-1B2 (ALPHA2), and a regulatory dimer of PAFAH1B1 (LIS1) (Hanahan, 1986; Kornecki and Ehrlich, 1988; Koltai et al., 1991a,b; Chao and Olson, 1993; Stafforini et al., 2003; Bazan, 2005; Escamez et al., 2012). *Lis1* is a gene strongly expressed in the cortical plate during embryogenesis and at postnatal stages in the hippocampus, neocortex and cerebellum (Reiner et al., 1995). Genetic variations in the lissencephalic critical region (17p13.3) around *LIS1* gene, have been observed in schizophrenia patients. Previous studies of our group have shown that patients showed a greater tendency toward genetic variations of Lis1 markers (Tabarés-Seisdedos et al., 2006, 2008). Moreover, variations of *LIS1* expression have been reported in the dorsolateral prefrontal cortex of patients with schizophrenia (Lipska et al., 2006) and functional alterations of LIS1 protein interactive complex in brain development are relevant to the pathogenesis of schizophrenia and related psychiatric illness (Bradshaw et al., 2011).

In this study, we analyzed the effect that deleting the first coding exon of the *Lis1* gene has on the distribution of GABAergic interneurons in the young-adult stages of *Lis1/sLis1* mutant mice. We found a reduction of GABAergic interneurons in both the hippocampus and Anterior Cingulate area (ACA) in *Lis1/sLis1* mutant mice compared to control animals. In addition, the immunohistochemistry of the cFOS protein was analyzed to evaluate neuronal activity in different brain areas. Furthermore, we explored the activation of GABAergic interneurons in *Lis1/sLis1* mice. To do this, we analyzed the expression of cFOS in GABAergic interneurons in cortical regions and the hippocampus. Finally, behavioral studies demonstrate the dysfunction of mutant mice in terms of spontaneous locomotion and cognitive tests.

Our results suggest that this mutation in the *Lis1* gene could cause changes associated with some SZ endophenotypes.

## Materials and Methods

### Animals

All mutant *Lis1/sLis1* mice had an ICR genetic background. The *Lis1/sLis1* transgenic line has been described previously (Cahana et al., 2001). For genotyping, PCR was performed as described in Cahana et al. (2001), using one set of primers to detect the gene codifying the shorter protein (sLIS1): Mlis1 5′-GGT GGC AGT GTT GAG ATG CCT AGC C-3′ and Mlis1 5′-GCA TTC CTG TAA TCC AGT ACC TGG-3′ (Supplementary Figure 1). The 67-green fluorescent protein (*Gad67-Gfp*) line was described by Tamamaki et al., 2003. The *Gad67-Gfp* × *Lis1/sLis1* strain was generated by crossing heterozygous *Lis1/sLis1* mice with *Gad67-Gfp* mice.

The animal experiments complied with the Spanish and European Union laws on animal care in experimentation (Council Directive 86/609/EEC). To stage the animals, we took the vaginal plug formation date as embryonic day 0.5 (E0.5). To fix adult brains from postnatal day 30 (P30) or 60 (P60) mice, the animals were anesthetized with isoflurane (Zoetis, United Kingdom), perfused transcardially with phosphate-buffered saline (PBS, pH7.4), followed by 4% paraformaldehyde (PFA) in PBS. The brains were subsequently removed and postfixed overnight at 4°C in the same fixative.

Some animals were selected for P60 behavior tests. An equal number of males and females were used in each experiment.

### Immunohistochemical Staining

The animals were processed for immunohistochemistry staining to quantify the three subtypes of GABAergic interneurons (PV+, CB+, and CR+ cells), glutamic acid decarboxylase (GAD67) and cFOS. After perfusion, the brains were removed, postfixed in the same fixative overnight and embedded in 4% agarose in PBS, and transversely sectioned into 70 μm-thick sections using a Vibratome (Leica). The brain slices were then rinsed with phosphate buffer solution containing 0.075% Triton X-100 (PBST) and processed for immunohistochemistry staining. For cFOS immunohistochemistry, the brains were soaked in 10, 20, and 30% sucrose solution at 4°C in PBS, and coronal sections (40-μm thick) were cut using a cryostat (HM525, Microm/Thermo Fisher Scientific, Waltham, MA, United States). The vibratome sections were incubated with 0.09% hydrogen peroxide (H_2_O_2_) for 30 min, rinsed with phosphate buffer solution containing 0.075% Triton X-100 (PBS-T). Next, the tissue was incubated with Goat Serum (GS) (blocking solution) for 1 h to avoid any non-specific antigen binding. The sections were then incubated overnight at 4°C with the proper primary antibodies diluted in EnVision FLEX Antibody Diluent (DAKO, Denmark). The antibodies used were: anti-green fluorescent protein (GFP) chicken polyclonal antibody (1:500; Aves Labs, Inc.,Tigard, Oregon); anti-calbindin D-28k (CB) rabbit polyclonal antibody (1:2000; Swant, Bellizona, Switzerland); anti-calretinin (CR) rabbit polyclonal antibody (1:2000; Swant, Bellizona, Switzerland); anti-parvalbumin (PV) polyclonal antibody (1:2000; Swant, Bellizona, Switzerland); and anti-cFOS rabbit polyclonal IgG (1:400; Calbiochem). The next day, the sections were rinsed three times at room temperature and incubated with the biotinylated secondary antibody for 1 h.

Afterward, the sections were washed with PBS-T and incubated with Avidin–Biotin Complex for 1 h (1:300; ABC kit, Vector Laboratories CA-94010). For the colorimetric detection (black), the tissue was incubated with 1% 3,39-Diaminobenzidine (DAB; Vector Laboratories SK-4100), 0.025% ammonium nickel sulfate hexahydrate, and 0.0018% H_2_O_2_ in PBS).

In some cases, we performed double immunohistochemistry for anti-PV and anti-vesicular glutamate transporter 1 (VGLUT1) polyclonal guinea pig antibody (1:4000; Millipore, Temecula, CA, United States) or anti-cFOS rabbit and anti-GFP. For this, the samples were processed for immunofluorescence staining (free Triton X-100). For this immunostaining, the sections were incubated with the appropriate secondary antibody for 1 h, after being incubated overnight with either anti-parvalbumin and anti-VGLUT1 or anti-cFOS and anti-GFP. The secondary antibodies used were: Alexa Fluor 488 donkey anti-rabbit IgG 1:500 (Molecular Probes/Invitrogen#A11055) and Alexa Fluor 488 goat anti-chicken IgG (A11039/Molecular Probes), and donkey anti-guinea pig IgG 1:200 (Biotium, Jackson Inmuno Research) or goat anti-rabbit IgG 1:300 (BA-9200/Vector) followed by Cy3-Streptavidin 1:500 (PA43001/Amersham).

Next, the sections were washed with PBS-T RT and the nuclei were counterstained with 4′,6-diamidino-2-phenylindole (DAPI, Molecular Probes/Invitrogen) diluted 1:1000 in PBS for 10 min and the sections were then washed in PBS. Finally, the sections were mounted on glass slides using 10:1 Mowiol (Calbiochem)-NPG (Sigma).

### Microscopy and Statistical Analysis

Images were acquired using optical (Leica CTR6000) and confocal (Leica TCS SPE) microscopes, and the images were used to make immunopositive cell counts. To quantify the number of cells, we counted four sections per animal, in a parallel series of sections stained using different markers; we used Image-J software (NIH, United States) for cell counting. The data used for statistical analysis was the mean of the four sections counted. To quantify VGLUT1+ staining on PV-interneurons we selected two sections and five interneurons in each section (see Figure 3 below). On the GFP+ channel (PV+ neurons) we chose a segment of a dendrite in each selected neuron, and then we measured the area of that segment and the area of red boutons/clusters placed in contact with that dendrite segment. The total area of the dendritic segments selected was the same in WT and mutant sections and we measured the% of dendritic area covered by red stained boutons; these measurements were made with Image-J in a blind manner.

We focused on three mPFC subregions: the ACA, the prelimbic cortex (PrL) and infralimbic cortex (IL); we studies also the hippocampal region and the perirhinal (PERI), entorhinal (ENT), ectorhinal (ECT) cortices in both the right and left hemispheres using at least four different sections for each brain area. To identify these regions, the Allen Brain Atlas^1^ was used as a guide. The average of these determinations for each section was defined as the number of immunopositive cells within a specified area in the individual brain. The average of these values among different sections of an individual brain was used for statistical analysis. In all cases, the data are expressed as the mean ± standard error of the mean (SE). Statistical differences between *Lis1/sLis1* mice and control littermates were determined using the Student’s *t*-test. The Mann–Whitney *U* test was used to compare differences between two independent groups when the dependent variable was not normally distributed or when their variances were not equal. To perform statistical analysis Sigma Plot software was used. A value of *p* < 0.05 was considered statistically significant. Significance levels were set to: ^∗^*p* < 0.05, ^∗∗^*p* < 0.01 and ^∗∗∗^*p* < 0.001.

### Behavioral Tests

#### Locomotor Activity

For the Open Field Test, the spontaneous activity of the mice was assessed by monitoring their activity in an open field (SMART VIDEO TRACKING Software, Panlab, Barcelona, Spain). The apparatus consisted of a square plexiglass box (50 cm wide × 50 cm long × 40 cm high). The mice (P60) were placed, individually, in the center of the apparatus, and left undisturbed for 10 min. The cage was thoroughly cleaned with 75% ethanol between each test to eliminate any olfactory cues. The total ambulatory distance was analyzed.

To record voluntary wheel-running activity, each cage was equipped with a running wheel (Med Associates Inc., Vermont, United States) at P60. The animals had constant voluntary access to the running wheel. The data was recorded with a standard computer using the Wireless Running Wheel Interface System (Med Associates Inc., Vermont, United States). The number of wheel revolutions each minute were recorded over a 72-h period. From the raw data, the total revolutions, day/night revolutions, and the 12-h revolution average were calculated using Excel 2016 (Microsoft Corporation, Washington, United States).

#### Novel Object Recognition Test

The Novel Object Recognition Test (NOR) (*n* = 12 for each group; P60) was used to assess non-spatial memory in a box (50 cm wide × 50 cm long × 40 cm high). After a 30 min habituation period the previous day, the behavior was recorded using a camera equipped with a computer-assisted data acquisition system (Smart, PANLAB, Spain), in both training and retention sessions. Two novel objects were symmetrically placed 15 cm from the walls during the training session. Each mouse explored for 5 min. Exploration of the objects was defined by the time a mouse spent with its head facing the objects within 1 cm of them, or if it was touching or sniffing them. The objects and box were cleaned with 75% ethanol after the training session. The retention tests were carried out 5 min after the training session, and one of the objects used during training was replaced with a novel object. Each mouse was then allowed to freely explore for 5 min. Exploratory preference was calculated by the ratio of the time spent exploring the novel object over the total time spent exploring the two objects.

## Results

### GABAergic Signaling

Data published to date has revealed the potential role of cortical GABAergic deficits in the symptoms and pathology observed in SZ patients (Lewis et al., 2012; Nakazawa et al., 2012; Stansfield et al., 2015; Li J.-T. et al., 2016). To study the GABAergic system in *Lis1/*s*Lis1* mutant mice, we quantified the number of PV, CR, CB and GAD67-positive neurons in the mPFC and hippocampus (Hi) of mice brains at 30 days (P30).

#### Reduction of Number of Cells Expressing GAD67 Is Observed in the Anterior Cingulate Area and Hippocampus in *Lis1/sLis1* Mutant Mice

**FIGURE 1:**
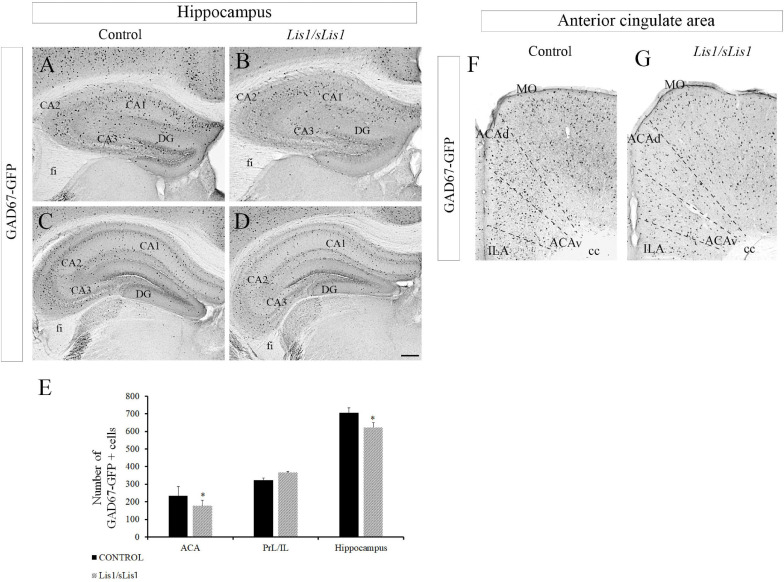
Study of GABAergic interneurons in the hippocampus and anterior cingulate area in *Gad67-Gfp* × *Lis1/sLis1* and *Lis1/sLis1* adult mice. Photomicrographs of coronal sections taken through the hippocampal area **(A–D)** and ACA **(F,G)**, processed using immunoperoxidase staining for GAD67- anti-green fluorescent protein (GAD67-GFP) chicken polyclonal antibody. **(A–D)** There were fewer GFP-positive cells in the hippocampus of *Lis1/sLis1* mice compared with adult controls. **(E)** Histogram indicating the average number of GFP+ cells ±SE in the ACA, PrL/ILA and hippocampus. **(F,G)** The number of GFP-positive cells in the anterior cingulate area of *Lis1/sLis1* mice was decreased compared to adult controls. For all panels scale bar is 200 μm.

The number of cells expressing GABA-synthesizing enzyme glutamic acid decarboxylase 67 (GAD67), responsible for most GABA synthesis, was studied in *Lis1/sLis1* mutant and control mice by using the mutant strain *Gad67-Gfp* × *Lis1/sLis1*. GAD67-positive cells were detected through GFP immunohistochemistry at P30. The distribution of GAD67+ interneurons was studied in the hippocampus and mPFC (Figure 1). A decrease in the number of interneurons expressing this enzyme was observed in the hippocampus of the *Lis1/sLis1* mutant mice (Figures 1A–E). GAD67 reactivity was noted in scattered cells of all the CA1–CA3 layers and, occasionally, in cells of the dentate gyrus (DG) in control mice (Figures 1A,C). The analysis of the *Lis1/sLis1* mutant hippocampus showed that the number of cells expressing GAD67 was reduced in the entire hippocampal area (Figures 1B,D; *n* = 4; controls, 705 ± 28; *Lis1/sLis1*, 623 ± 26; *p* < 0.05, Student’s *t*-test). These differences become more evident in the CA3 area, where a severe reduction of GAD67+ cells was observed (Figure 1D). The mPFC is subdivided into three main parts: the anterior cingulate (ACA), prelimbic (PrL), and infralimbic (IL) areas (Tavares and Corrêa, 2006; Vertes, 2006). Although no differences were observed in the number of GAD67-positive interneurons in the infralimbic (IL) and prelimbic (PrL) areas, a reduction of the number GAD67+ interneurons was detected in the ACA (*n* = 4; Figures 1F–H; *n* = 4; controls, 235 ± 50; *Lis1/sLis1*, 176 ± 32; *p* < 0.05, Student’s *t*-test).

#### Fewer PV+ Cells in the Anterior Cingulate Area of *Lis1/sLis1* Mutant Mice

**FIGURE 2:**
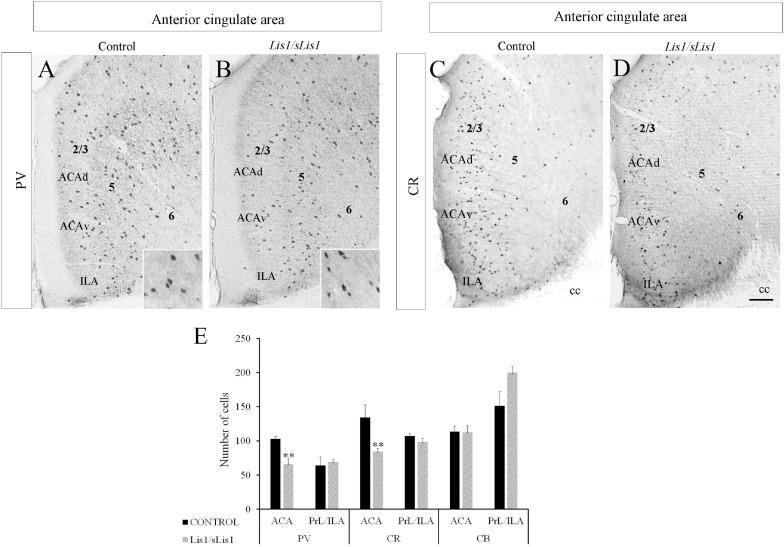
Study of GABAergic interneurons in the anterior cingulate area in control and *Lis1/sLis1* mutant mice in adults. **(A–D)** Photomicrographs of coronal sections taken through the anterior cingulate area and processed by immunoperoxidase staining for parvalbumin (PV) and calretinin (CR) using single immunostaining. There were fewer PV-positive cells in the anterior cingulate area of *Lis1/sLis1* mice than the controls **(A,B)**. A higher magnification of PV+ cells is inserted in the bottom right. CR immunostaining demonstrates a decrease in CR-positive neurons in *Lis1/sLis1* mice versus adult control littermates **(C,D)**. **(E)** Histogram indicating the average ±SE in WT and *Lis1/sLis1* mutant mice of PV+ cells, CR+ cells and CB+ cells in the ACA and PrL/IL. For all panels the scale bar is 200 μm. For inserts in **(A,B)**, scale bar is 50 μm.

Alterations of PV-expressing interneurons have been implicated in many neuro-psychiatric diseases, including schizophrenia (Flames et al., 2004; Fisahn et al., 2009; Fazzari et al., 2010; Ting et al., 2011; Huang et al., 2021). Accordingly, we analyzed the distribution of PV-positive interneurons in *Lis1/sLis1* mutant and control mice in the PrL, IA, and ACA areas (Figure 2). No differences were observed in the number of PV-positive interneurons in the PrL and IL areas (*n* = 4, average number of PV-positive interneurons ± SE: controls, 63.9 ± 7.54; *Lis1/sLis1*, 68.9 ± 3.28). We detected a significant decrease of PV interneurons in the ACA of *Lis1/sLis1* mutant mice compared to control animals (*n* = 4; Figures 2A,B; *n* = 4; average number of PV+ neurons ± SE: controls, 103 ± 3,30; *Lis1/sLis1*, 65 ± 8,70; *p* < 0.01, Student’s *t*-test). That reduction of PV+ cells was prominent in layers 2/3 and 5, where they are most abundant, whereas differences in layer six were less marked (Figure 2B). The values shown correspond to the mean ± SE (*n* = 4; Figure 2E). No significant differences in PV interneurons were observed in the hippocampus (*n* = 4, average number of PV-positive interneurons ± SEM: controls, 104 ± 6.77; *Lis1/sLis1*, 110 ± 6.15).

#### Decreased CR+ Cells in the Anterior Cingulate Area of *Lis1/sLis1* Mutant Mice

As a continuation of the study of GABAergic populations of interneurons, we examined the distribution of CR and CB positive interneurons in the mPFC of control and *Lis1/sLis1* mutant mice. In control mice, we observed CR+ interneurons distributed throughout the ACA, most frequently being found in layer 2/3, whereas in layers 5 and 6 less CR-positive interneurons were marked (Figures 2C,D). In accordance with our previous results on PV interneurons, we observed no differences in the number of CR-positive interneurons in the PrL and IL areas of the mPFC (*n* = 4, average number of CR-positive interneurons ± SE: controls, 107 ± 4.05; *Lis1/sLis1*, 98 ± 5.46). Nevertheless, a decrease in CR interneurons was observed in ACA in *Lis1/sLis1* mutant mice (Figures 2C,D). The values shown correspond to the mean ± SE (*n* = 4; Figure 2E; *n* = 4 average number of CR+ neurons ± SE: controls, 134 ± 18; *Lis1/sLis1*, 84 ± 4; *p* < 0.01, Student’s *t*-test). No significant differences were observed for CB-positive interneurons in any of the mPFC regions (*n* = 4, average number of CR-positive interneurons ± SE: controls, 114 ± 8.05; *Lis1/sLis1*, 112 ± 9.70). The analysis of the hippocampus in *Lis1/sLis1* mutant mice revealed no differences in CB- or CR-expressing interneurons between mutant and control mice (*n* = 4, average number of CR-positive interneurons ± SE: controls, 109 ± 8.50; *Lis1/sLis1*, 105 ± 8.72; *n* = 4, average number of CB-positive interneurons ± SE: controls, 163 ± 32.29; *Lis1/sLis1*, 169 ± 17.60).

#### Decreased VGLUT1+ Terminals Contacting PV+ Interneurons in the Hippocampus of *Lis1/sLis1* Mutant Mice

**FIGURE 3:**
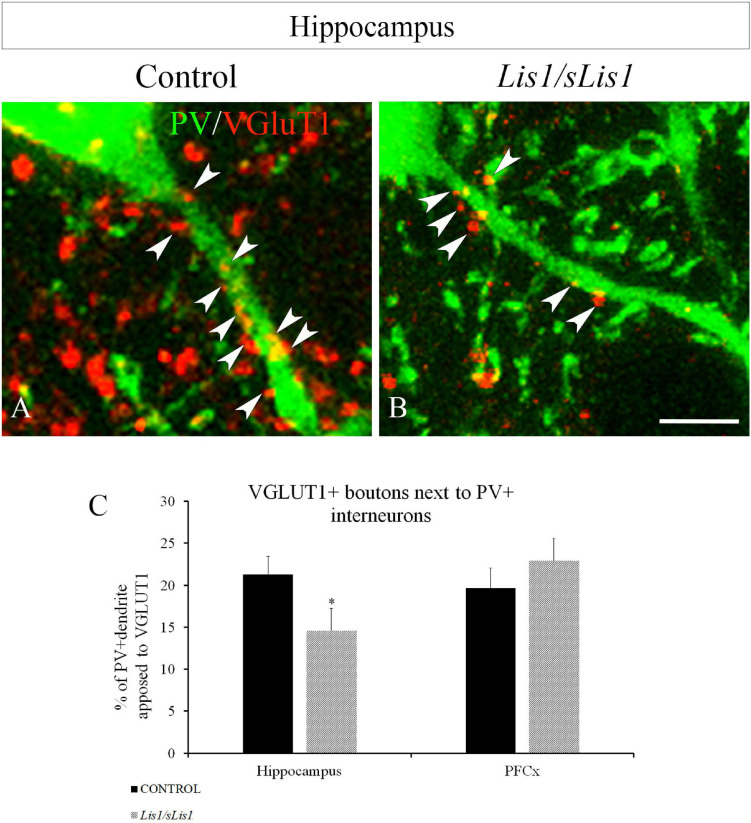
VGLUT1+ boutons next to PV+ interneurons in the hippocampal area (all CA1-3 and DG) in control and *Lis1/sLis1* mutant mice. **(A,B)** PV+ interneurons displayed in green and VGLUT1 boutons in red in control **(A)** and *Lis1/sLis1* mutant mice **(B)**. **(C)** The histogram shows the percentage cover of hippocampal and prefrontal PV+ cells with VGLUT1 boutons in *Lis1/sLis1* and control mice. The % of PV+ dendrite opposed to VGLUT1 in the hippocampus is reduced in *Lis1/sLis1* mutant mice compared to controls. Scale bar 5 μm in **(A,B)**.

Previous studies have reported decreased excitatory terminals in cortical interneurons in SZ patients (Chung et al., 2016) as well as in mouse models displaying a SZ-like phenotype (Del Pino et al., 2013). The reduced number of PV+ interneurons and the lowered excitatory drive to PV+ cells (Coyle et al., 2012; Gonzalez-Burgos et al., 2015; Chung et al., 2016) have been proposed as the neural substrate for cognitive dysfunction in SZ. Therefore, we first analyzed the distribution of PV-positive interneurons in the hippocampal areas of *Lis1/sLis1* mutant and control mice. As we have described above, no differences were observed in the number of PV-positive interneurons in the hippocampus. Next, we quantified the excitatory synapses contacting PV+ interneurons. To do this, we stained the vesicular glutamate transporter 1 (VGLUT1) by immunohistochemistry to detect excitatory presynaptic contacts with PV+ interneurons in *Lis1/sLis1* mutant mice and in their control littermates. We quantified the area of VGLUT1 boutons contacting PV+ interneurons in the hippocampal region and in the mPFC of *Lis1/sLis1* mutant and control mice, using the Image J software. We observed a decrease in the area covered by VGLUT1 boutons in PV+ cells in both the CA1-3 and DG regions of *Lis1/sLis1* mutants compared to controls (Figures 3A–C; *n* = 4, average of% of PV+ dendrite opposed to VGLUT1 ± SE: controls 21.3 ± 2.1%; *Lis1/sLis1*, 14.6 ± 2.6%, *p* < 0.05, Student’s *t*-test). However, no significant differences were observed in the mPFC (*n* = 4, average of% of PV+ dendrite opposed to VGLUT1 ± SE: controls 19.7% ± 2.3%; *Lis1/sLis1*, 22.9% ± 2.6%). These results demonstrate that hippocampal PV interneurons of CA1-3 and DG receive a reduced number of excitatory synapses in *Lis1/sLis1* mutant mice.

### Increased Number of Positive Cells for cFOS in the mPFC, Hippocampus and Other Related Cortical Areas in *Lis1/sLis1* Mutant Mice

**FIGURE 4:**
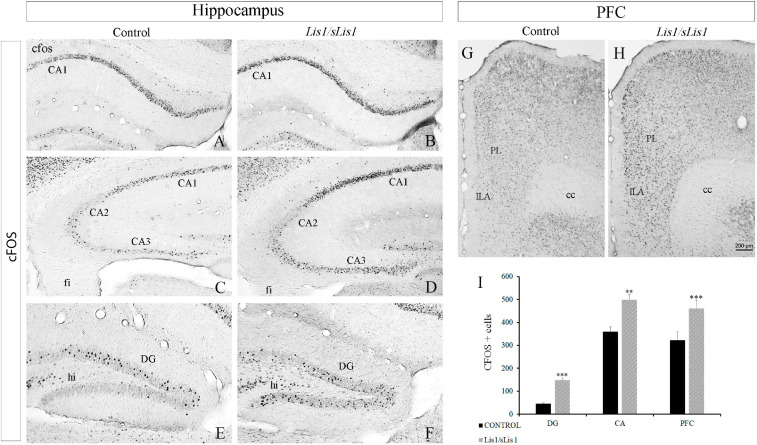
Cells expressing cFOS in the hippocampus and PFC in *Lis1/sLis1* mutant mice. **(A–H)** Coronal sections are immunostained with anti-cFOS antibody in the hippocampus **(A–F)** and prefrontal cortex **(G,H)** at P60. There was an increase in cFOS+ cells in the CA **(A–D)** and DG **(E,F)** regions of the hippocampus in *Lis1/sLis1* mutant mice. Moreover, *Lis1/sLis1* PFC also showed an increased number of cells expressing cFOS **(G,H)**. **(I)** The histogram represents the statistically significant increase in cFOS-positive cells in the areas analyzed (DG, CA, and PFC). For all panels scale bar is 200 μm.

Proteins coded by immediate-early genes such as *cFOS*, are considered markers of neuronal activity in the central nervous system (Sagar et al., 1988; Herdegen et al., 1995; Kontkanen et al., 2002). Moreover, it has been established that the number of cFOS-expressing neurons increases in animal models of schizophrenia (Celada et al., 2013; Hervig et al., 2016; Calovi et al., 2020). Here, we analyzed the distribution pattern of cFOS immunopositive cells in the hippocampal areas, mPFC, PERI, ECT, and ENT of both control and *Lis1/sLis1* mutant mice. To this end, we quantified the number of cells expressing cFOS. We counted cFOS positive cells at P60 in control and *Lis1/sLis1* mutant mice that had been exposed to the same basal environmental stimuli in the animal facility (face-to-face cages in the same room). In the hippocampus of control young-adult mice most of the cFOS protein was expressed in the granular cell layer of the DG and the pyramidal cell layer of CA1–CA3. cFOS was also detected, but at lower densities, in the DG molecular layer (hilus), and in the stratum oriens of the CA1–CA3 (Figures 4A,C,E). We detected a significant increase in cFOS immunopositive cells in CA1–CA3 regions (Figures 4B,D), as well as in the dentate gyrus (Figure 4E) in the hippocampus of Lis1/sLis1 mutant mice compared to control mice (*n* = 4; average number of cFOS+ cells in CA ± SE: controls, 359 ± 20, *n* = *4*, *Lis1/sLis1*, 498 ± 22, *n* = *4*; *p* < 0.01, Student’s *t*-test; average number of cFOS+ cells in DG ± SE: controls, 45 ± 4, *n* = *4*, *Lis1/sLis1*, 148 ± 9, *n* = *4*; *p* < 0.001, Student’s *t*-test). Moreover, we analyzed cells positive for cFOS in both the suprapyramidal (DGsp) and infrapyramidal (DGip) blades of the DG. In control mice, cFOS positive cells were mainly observed in the DGsp, whereas fewer cFOS positive cells were seen in the DGip (Figure 4E), suggesting a different expression pattern of cFOS protein in the two DG blades. Strikingly, this difference in neuronal activity between DGsp and DGip was not observed in the DG of *Lis1/sLis1* mice. The DGip of the DG in *Lis1/sLis1* mutant mice there was a greater number of cells expressing cFOS compared to the control mice (Figures 4E,F,I).

Subsequently, we analyzed cFOS-positive cells in the mPFC and observed that this area also presented more cFOS-positive cells in *Lis1/sLis1* mice than the control animals (Figure 4G–I; *n* = 4; average number of cFOS+ cells in mPFC ± SE: controls, 322 ± 36, *n* = *4*, *Lis1/sLis1*, 460 ± 37, *n* = *4*; *p* < 0.001, Student’s *t*-test).

**FIGURE 5:**
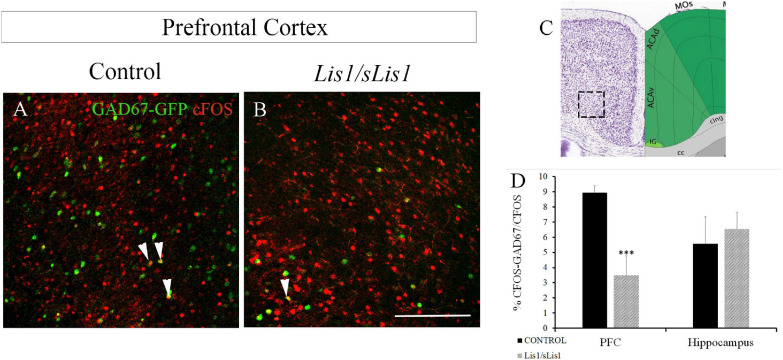
Study of the number of co-expressing cFOS and GAD67 cells at P60 in the mPFC of control and *Lis1/sLis1* mutant mice. **(A,B)** The number of cells co-expressing cFOS and GAD67 in the mPFC area of *Lis1/sLis1* mutant mice was decreased compared to control mice. **(C)** The image was taken from the Allen Brain Atla**s** showing the approximate area of panels **(A,B)**, where the cells were counted. **(D)** Histogram indicating the average number of co-expressing cFOS and GAD67 cells ±SE in the young-adult mPFC and hippocampus. Scale bar is 200 μm.

In addition, we analyzed the neuronal activity of GAD67+ interneurons. To this end, we quantified the number of cells co-expressing cFOS and GAD67 at P60 in control and *Lis1/sLis1* mutant mice (Figure 5). We analyzed the number of GAD67+ interneurons that were immunopositive for cFOS in both the mPFC and hippocampal areas. We detected fewer cells co-expressing cFOS and GAD67 with respect to the total number of cFOS positive cells, in the mPFC area of *Lis1/sLis1* mutant mice compared to control mice (Figures 5A–D; *n* = 4; average number of% cFOS-GAD67/cFOS cells ± SE: controls, 8.95 ± 0.45; *Lis1/sLis1*, 3.48 ± 1.28, *p* < 0.001, Student’s *t*-test). No significant differences were observed in the number of cells co-expressing cFOS and GAD67 when the hippocampus was analyzed (n = 4, average number of% cFOS-GAD67/cFOS cells ± SE: controls, 5.56 ± 1.79; *Lis1/sLis1*, 6.56 ± 1.11). However, when the number of cells co-expressing cFOS and GAD67 with respect to the total number of GAD67 positive cells was studied in the mPFC area of *Lis1/sLis1* mutant mice compared to control mice, no significant differences were detected suggesting that interneuron excitability remains intact.

**FIGURE 6:**
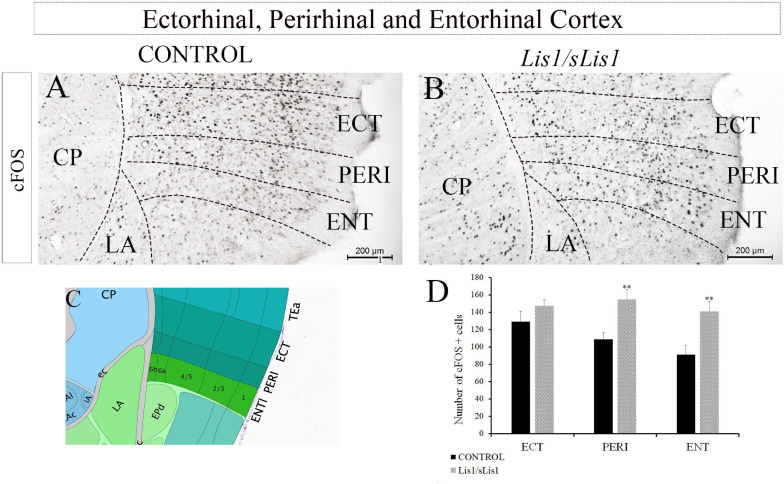
Analysis of cells expressing cFOS in other hippocampus-related cortical regions. **(A,B)** The entorhinal, perirhinal, and ectorhinal cortices were assessed. The number of cells positive for cFOS was quantified in these areas and the perirhinal and entorhinal areas of the *Lis1/sLis1* mutant mice presented a larger number of cells expressing cFOS **(B)** than the controls **(A)**. No significant differences in the ectorhinal cortex were detected between the *Lis1/sLis1* mutant mice and controls. **(C)** The image was taken from the Allen Brain Atla**s** showing the approximate area of panels **(A,B)**. **(D)** The histogram shows these results in the areas analyzed (ECT; PERI, and ENT). Scale bar is 200 μm.

Hippocampal functions such as episodic memory and spatial orientation, are associated with other brain areas like the perirhinal (PERI), entorhinal (ENT), and temporal associative cortices, as well as the prefrontal cortex (Aggleton and Brown, 1999). Moreover, ENT axons are the most significant cortical input to the hippocampus (Warburton and Brown, 2010). We therefore decided to analyze whether the functional activity of these areas is affected in *Lis1/sLis1* mutant mice. To this end, cellular activity in the PERI, ECT, and ENT cortices was also analyzed through cFOS immunohistochemistry (Figure 6). We observed an increment of cFOS+ cells in PERI and ENT in *Lis1/sLis1* mutant mice compared to the controls (*n* = 4; Figures 6A–D average number of cFOS+ cells in the perirhinal area ± SE: controls, 108 ± 8, *n* = *4*, *Lis1/sLis1*, 154 ± 11, *n* = *4*; *p* < 0.01, Student’s *t*-test; average number of cFOS+ cells in the entorhinal area ± SE: controls, 91 ± 10, *n* = *4*, *Lis1/sLis1*, 140 ± 11, *n* = *4*; *p* < 0.01, Student’s *t*-test).

In contrast, no differences were observed when ECT was analyzed (Figures 6A–D; *n* = 4, average number of cFOS+ cells in the ectorhinal area ± SE: controls, 129 ± 11; *Lis1/sLis1*, 148 ± 6).

Our results show a significant increase in the number of cells expressing the cFOS protein in certain areas of the *Lis1/sLis1* brain, which mimic those described in postmortem brains of SZ patients and schizophrenia-like phenotype mouse models (Dragunow and Faull, 1990; Abdel-Naby Sayed et al., 2001; Keilhoff et al., 2004).

### Behavioral Alterations in *Lis1/sLis1* Mutant Mice

**FIGURE 7:**
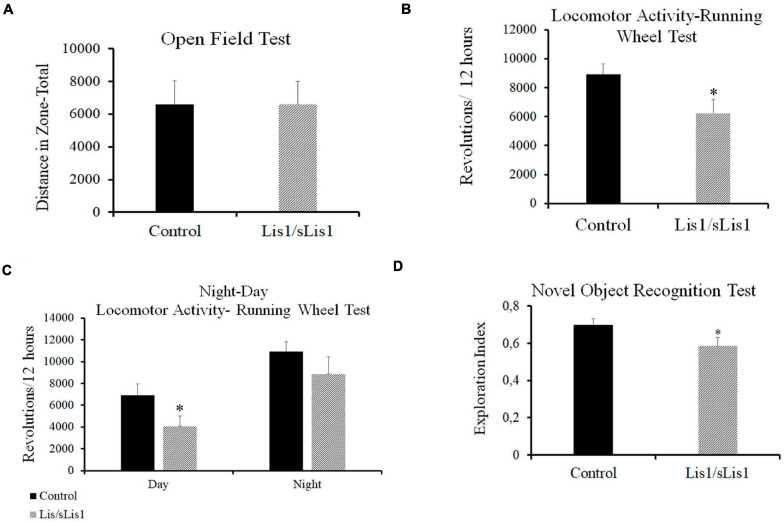
Behavioral alterations in *Lis1/sLis1* mutant mice. **(A)** Ambulatory locomotion was analyzed using an Open Field Test, but no behavioral differences were observed in *Lis1/sLis1* mutant mice compared to controls. **(B)** Spontaneous locomotor activity was analyzed through a Running Wheel Analysis. The histogram shows a reduction in the total activity averaged over 12 h in *Lis1/sLis1* mutant mice. **(C)** Night-Day locomotor activity analysis showing a statistically significant trend toward less activity during the day. No differences were detected during the nighttime period. **(D)** The Novel Object Recognition Test was used to study memory impairment in *Lis1/sLis1* mutant mice. The histogram shows a lower exploration index when the mice were exposed to a new object together with a familiar object.

To analyze the functional effects of the observed cortical alterations, behavioral analyses were performed. Firstly, ambulatory locomotor activity was assessed through the Open Field Test. The total distance traveled by the *Lis1/sLis1* group was not significantly different to the control group (*n* = 12 for each group; Figure 7A).

Sleep disorders and circadian rhythm alterations have been described in SZ patients (Wulff et al., 2011). Furthermore, studies in animal models have suggested that schizophrenia-like phenotype mouse models display circadian rhythm disruption (Tam et al., 2015). We therefore studied spontaneous locomotor activity by means of a Running Wheel Analysis over a 72-h period. This test analyzes both circadian rhythms and locomotor activity. We observed decreased voluntary activity in the *Lis1/sLis1* mutant mice compared to the controls. Mutant mice made significantly fewer rotations than controls when considering a 12 h average (Figure 7B; *n* = 12 for each group, average number of revolutions/12 h ±SE: controls, 8909 ± 737, *n* = *12*, *Lis1/sLis1*, 6213 ± 949; *p* < 0.05, Student’s *t*-test). We then wanted to study whether these differences were due to a general decrease in activity and if there were differences between daytime and nighttime activity. Firstly, we analyzed the rest-activity rhythms in the control and mutant mice, respectively. Our results showed that both the controls and mutants presented statistically significant differences between their night vs. day activity (*n* = 12; *p* < 0.01 for control mice and *p* < 0.001 for *Lis1/sLis1* mutant mice). As expected, there was an increase in spontaneous locomotor activity at night in both control and mutant mice (Figure 7C). Next, we analyzed potential night-day locomotor activity differences between control and *Lis1/sLis1* mutant mice. We observed more rest time during the day of *Lis1/sLis1* mutant mice, differences were statistically significant when compared with control mice. On the other hand, no significant differences in spontaneous locomotor activity were observed during the night (Figure 7C; *n* = 12 for each group, average number of revolutions/12 h ±SE. Day: controls, 6901 ± 1041, *Lis1/sLis1*, 4066 ± 923; *p* < 0.05, Student’s *t*-test; Night: controls, 10918 ± 887, *Lis1/sLis1* 8866 ± 1583).

Recognition memory deficits in SZ are an important cognitive symptom suffered by patients (Green et al., 2000; Tam et al., 2015). Memory deficits have also been reported in genetic SZ mouse models such as *Nrg1*-deficient mice (Harrison and Owen, 2003; Duffy et al., 2010; Pei et al., 2014) and in pharmacological, lesion and developmental SZ rodent models (Lyon et al., 2012). As our previous histological results showed that *Lis1/sLis1* mutant mice display quantitative and qualitative alterations of cell expressing cFOS in the hippocampus and in perirhinal and entorhinal cortices, we studied the possible existence of memory affectation. To this end, we performed the Novel Object Recognition (NOR) task. Impaired recognition memory was seen in *Lis1/sLis1* mice in this task. Mutant mice showed a significantly lower exploration index regarding the novel object compared to the overall exploration than wild-type mice (Figure 7D, *n* = 12 for each group; average of the exploration index ± SE: controls, 0.7 ± 0.03, *n* = *12*, *Lis1/sLis1*, 0.58 ± 0.045; *p* < 0.05, Student’s *t*-test). No differences were observed between *Lis1/sLis1* mutant mice and controls in the training session (data not shown).

## Discussion

### GABAergic Signaling in *Lis1/sLis1* Mutant Mice

The role of the *Lis1* gene in the development and function of the GABAergic system has been studied using a hypomorphic mutation of the *Lis1* allele (*Lis1/sLis1*) (Cahana et al., 2001, 2003), where the first coding exon from the *Lis1* gene has been deleted. Homozygous mice are not viable, but the study of heterozygous mice has revealed an abnormal neuronal morphology, cortical dysplasia, and enhanced excitability. Previous studies in our laboratory have demonstrated structural differences in cortical development (Cahana et al., 2001, 2003), the functional properties of cortical circuits (Valdés-Sánchez et al., 2007) and the basal forebrain, and alterations in septohippocampal projections in *Lis1/sLis1* mutant mice (Garcia-Lopez et al., 2015). In addition to cortical morphology defects, we have linked variation in *LIS1* expression to human psychosis (Tabarés-Seisdedos et al., 2008). Here, we have examined *Lis1/sLis1* mutant mice to study how the altered *Lis1* gene expression might cause deficits associated with the pathophysiology of SZ.

We found that deleting the first coding exon of the *Lis1* gene affects the GABAergic cortical circuits. Many studies have related dysfunction of the GABAergic system with several cognitive impairments in SZ (Lewis et al., 2012; Sullivan and O’Donnell, 2012). Postmortem studies have shown decreased levels of mRNA from GABAergic markers, including glutamate decarboxylase 1 (GAD67) and parvalbumin (PV), with reduced PV expression being one of the most widely replicated findings in SZ (Akbarian et al., 1995; Guidotti et al., 2000; Fung et al., 2010; Gilabert-Juan et al., 2012; Glausier et al., 2014; Hoftman et al., 2015). Specifically, the mPFC and hippocampus seem to be the two main brain regions that present an altered GABAergic system in SZ (Heckers and Konradi, 2002; Heckers et al., 2002; Reynolds et al., 2004). Indeed, a significant reduction in the density of GABAergic inhibitory interneurons in the hippocampal region of schizophrenic brains has been previously reported (Benes and Berretta, 2001; Heckers and Konradi, 2002; Heckers et al., 2002). Moreover, studies of the hippocampus in SZ patients have reported a decreased population of the parvalbumin GABAergic interneurons (Zhang and Reynolds, 2002). In line with findings from studies in humans, deficits in GABAergic interneurons have been consistently described in different animal models of SZ (Jentsch and Roth, 1999; Thomsen et al., 2010; Ozdemir et al., 2012; Li J.-T. et al., 2016; Hauser et al., 2017). Our results demonstrate that the *Lis1/sLis1* hippocampus also shows a reduction in the number of cells expressing GAD67, in agreement with the observations made in other schizophrenia-like animal models. Interestingly, we observed a more severe reduction in CA3, which is innervated by ENT axons, either directly via the perforant path or indirectly from the dentate gyrus via the mossy fibers, the region with the most internal connectivity being in the hippocampal area (Amaral and Witter, 1989; Cherubini and Miles, 2015).

Several studies have also observed changes in other GABAergic interneuron subtypes, including CB, neuropeptide Y, somatostatin, vasoactive intestinal peptide, and cholecystokinin in the prefrontal cortex of SZ patients (Hashimoto et al., 2003, 2008; Fung et al., 2010, 2014). Furthermore, the anterior cingulate cortex (ACA) region of the brain presents an abnormal structure and function in SZ patients (Paus, 2001; Ridderinkhof et al., 2004; Rushworth et al., 2007). The ACA is a part of the mPFC that is involved in functions as diverse as associative learning, fear conditioning, and cognitive processes like attention. The functional heterogeneity of the ACA might explain the diversity of symptoms observed when this region is affected. These symptoms include apathy, inattention, dysregulation of autonomic function, and emotional instability, which are present in SZ (Picard and Strick, 1996; Andreasen et al., 1998; Vogt, 2005; Pietersen et al., 2007). To characterize our animal model with regard to the GABAergic system, we used GAD67, PV, and CR as protein markers. We found reduced numbers of GAD67+ interneurons in the ACA of *Lis1/sLis1* mutant mice compared to control animals. In addition, we detected a selective reduction in the density in the PV- and CR-expressing sub-population of GABAergic interneurons in the ACA. These findings are also consistent with previous studies using genetic models of SZ that reported decreased densities of PV-positive interneurons (Flames et al., 2004; Fisahn et al., 2009; Fazzari et al., 2010; Ting et al., 2011). Our mouse model, however, did not present a decrease in the number of PV+ interneurons in the hippocampus as has been widely observed in postmortem studies in humans and schizophrenia-like mouse models.

Other studies reported that lower PV levels or fewer excitatory synapses on PV+ neurons were associated with SZ sufferers, where no loss of PV neurons was observed in postmortem analysis (Chung et al., 2016). That work reported a 12% reduction in VGLUT1 puncta on PV+ cell bodies in SZ subjects, demonstrating fewer synaptic glutamatergic structures on PV+ neurons. Similar results have been reported for mutant mouse models with a schizophrenia-like phenotype (Fazzari et al., 2010; Del Pino et al., 2013). The decreased excitatory input into PV+ cells in the hippocampus that we observed may be altering the excitatory-inhibitory balance in the hippocampus of *Lis1/sLis1* mutant mice.

Our results therefore suggest that *Lis1* gene dysfunction might be responsible for the anomalies observed in the GABAergic system, which are also described in the pathophysiology of SZ.

Genetic studies have shown several susceptible genes in SZ pathogenesis, such as the neuregulin 1 (NRG1) and ErbB4 receptors (Williams et al., 2003; Stefansson et al., 2004; Harrison and Weinberger, 2005; Del Pino et al., 2013; Wakuda et al., 2015). The *LIS1* gene (Reiner et al., 1995) encodes a non-catalytic subunit of platelet-activating factor acetylhydrolase-1b (PAFAH1b) (Hattori et al., 1994), a brain-specific enzyme that inactivates PAF. *LIS1* is one of the main genes related to Type I lissencephaly, a serious human brain malformation caused by the abnormal formation of the brain during development, the mildest mutations of which lead to a predisposition to several mental disorders (Dobyns et al., 1993; Reiner et al., 1995; Kato and Dobyns, 2003; Barkovich et al., 2005; Wynshaw-Boris, 2007; Tabarés-Seisdedos et al., 2008). At prenatal stages, when interneurons are being generated, the PAFAH1B complex (LIS complex) consists mostly of ALPHA1 and LIS1 dimers. Later in development, at perinatal stages, *Alpha1* levels decreased and *Alpha2* levels increased, to generate new complex forms. At stages E14.5–E17.5, Lis1 is intensely expressed in both the VZ and SVZ layers in the pallium and in the subpallium (Escamez et al., 2012). sLIS protein is not capable of homodimerization and does not interact with LIS1 complex catalytic subunits (Cahana et al., 2001). It has been proposed that aberrant protein-protein interactions of sLIS1, could interfere in microtubules properties affecting cytoskeleton and causing defects in the cortical plate formation and in neuronal migration (Reiner, 2000). However, *Alpha1* transcript is expressed stronger in the subventricular zone (SVZ) of the ventral pallial region than in the dorsal and medial pallium suggesting a role in interneurons development. Interestingly, *Alpha1* expression is observed from early embryonic stages, with the highest peak at E14.5, until prenatal stages, and then it decreases. In *Lis1/sLis1* mutant mice lower values of *Alpha1* expression were found during embryonic development and an abrupt decline at P0 (Escamez et al., 2012). The highest peak stage coincided with the expansion peak of the tangential migration of tangential interneurons in mice (Marín and Rubenstein, 2003). The decrease of *Alpha1* gene expression together with *Lis1* gene mutation in *Lis1/sLis1* mutant mice could explain why interneurons are specially affected in *Lis1/sLis1* mutant mice. The defective protein-protein interaction of sLIS1 protein together with the reduced expression of ALPHA1, described above, could be the biochemical basis of the phenotype observed in *Lis1/sLis* mutant mice and could explain defects in interneuron development.

In addition, while mutation in other gene regions of *LIS1* have been associated to evident structural brain alterations described as lissencephalic spectrum disorders and associated to cerebral palsy and epilepsy (Pilz et al., 1998; Cardoso et al., 2002), deletion of the first coding exon shows a mild phenotype compatible with the histopathological data in SZ (Dobyns et al., 1993; Tabarés-Seisdedos et al., 2008).

It is well known that until late adolescence, the functional maturation of the GABAergic interneurons is not achieved, and it has been hypothesized that SZ could be associated with an alteration in the development of interneurons (Di Cristo, 2007; Hoftman and Lewis, 2011; Gonzalez-Burgos et al., 2015). Recently, it has been proposed that the maturation of all PV+ neurons in mouse PFC is rapid and almost completed by onset of puberty (Bitzenhofer et al., 2020). Thus, PV+ neuron maturation may contribute to the emergence of cognitive function primarily during prepubertal development.

### Cells Expressing cFOS in *Lis1/sLis1* Mutant Mice

For the last three decades, the cFOS protein has been used as a marker for transcriptional activity (Sagar et al., 1988), and cFOS immunohistochemistry allows the labeling of stimulated neurons. Previous studies using pharmacological mouse models for SZ have shown that phencyclidine (PCP) and related drugs increased cFOS expression levels in the mPFC and hippocampus (Näkki et al., 1996; Sato et al., 1997; Kargieman et al., 2007; Santana et al., 2011; Castañé et al., 2015; Calovi et al., 2020). Moreover, it has been reported that cFOS expression induced by PCP occurs in GAD67+ interneurons, specifically in the hippocampus, RSC, amygdala, and somatosensory cortex (Santana et al., 2011). Interestingly, cFOS induced by PCP in PV+ interneurons was detected in cortical areas such as the ventrolateral orbitofrontal cortex, RSC, and CA1, whereas cFOS induction in the motor cortex co-labeled with CB+ interneurons (Hervig et al., 2016). Thus, in this study we used cFOS immunohistochemistry to compare the activation degree of distinct brain regions in *Lis1/sLis1* mutant mice. As we observed an important alteration in the GABAergic system that could have been affecting the excitation-inhibition balance in several cortical areas in our mutant mice, we decided to assess the neuronal activation in the cortex. As expected, we observed increased cFOS positive cells in *Lis1/sLis1* mutant mice in the same areas where the number of interneurons was decreased. In fact, a significant increase in cFOS was found in the hippocampus, mPFC, ENT, and PERI compared to the control mice. These results suggest that *Lis1/sLis*1 neuronal dysplasia originates an imbalance in neuronal activity in *Lis1/sLis1* mutant mice, as has been reported in other SZ mouse models. The reduction in interneurons (less GAD67-positive neurons) may underlay the hyperactivity of glutamatergic (piramidal) neurons (cFOS overexpression). The ectopic activation and expression of cFOS has been proposed as a key factor responsible for the symptoms in SZ when PCP-related drugs are administered (Sharp et al., 1991; Post, 1992; Moghaddam et al., 1997). Indeed, increased cFOS expression has been suggested to play a role in negative symptoms observed in schizophrenia-like behavior (Abdel-Naby Sayed et al., 2001). On the other hand, some alleles of the *FOS* gene have been found to play a protective role, whereas others seem to be a risk factor for SZ, which has been connected to schizophrenia-related alterations in synaptic plasticity (Boyajyan et al., 2016). Interestingly, we observed higher cFOS activity in the DGsp of control mice, consistent with previous studies showing suprapyramidal-infrapyramidal gradients of neuronal activity in DG, which is not as functionally homogeneous as previously considered. Several observations have proposed that suprapyramidal (DGsp) and infrapyramidal (DGip) blades show different granule cell morphology and have asymmetric activity. Indeed, neurogenetic differences along the longitudinal (dorsal to ventral) and transverse (suprapyramidal and infrapyramidal) axes of the DG have been described (Jinno, 2011).

After a stimulus, DGsp is more quickly activated by entorhinal projections than DGip. The activation of DGsp is thought to occur together with inhibition to allow the DGsp to exceed DGip in response to the stimulation (Canning and Leung, 1997). Different levels of cFOS activity during spatial task performance have been described, with DGsp being more active during the task and DGip responding later (Scharfman et al., 2002; Chawla et al., 2005; Ramirez-Amaya et al., 2006; Marrone et al., 2012; Satvat et al., 2012; Gallitano et al., 2016). Interestingly, we found that the number of cFOS-positive cells in the infrapyramidal blade of the DG was greater in *Lis1/sLis1* mice compared to control mice, making the activity levels equivalent in both the DGsp and DGip of *Lis1/sLis1* mutants. Indeed, we found that whereas granule cells in the DGsp and DGip show different gradients of activation in control mice, these gradients are not present in the DG of *Lis1/sLis1* mutant mice, suggesting that there may be some dysfunction in terms of memory-related processes. Schmidt et al. (2012) described important anatomical and functional differences between DGsp and DGip blades that may be involved in pattern separation, and which are necessary to recognize small differences between similar stimuli and develop episodic memories (Schmidt et al., 2012).

These results suggest that the alteration of cFOS expression in DG granule cells disrupts activity patterns in DG circuits and, subsequently, causes cognitive alterations due to blade-dependent differences in excitability. This functional difference is not dependent on interneuron decreases, since these cells are scarce in DG. The *Lis1/sLis1* mouse shows spontaneous disruption of the normal DG activity pattern, probably as a consequence of altered connectivity, which may represent a new model for studying the functional significance of differences in inter-blade activity in episodic memory development by using specific experimental paradigms.

A deviation of VGLUT1 fibers in the GCL of *Lis1/sLis1* mutant mice was described in a previous study (Wang and Baraban, 2008). These authors suggest that these fibers could target either different compartments of postsynaptic cells or completely inappropriate postsynaptic targets. The increase in excitation of regions of the DG in *Lis1/sLis1* mutants could be in line with this alteration of VGLUT1 fibers.

### Behavioral Alteration in *Lis1/sLis1* Mutant Mice

The GABAergic system alteration and the increased cFOS-positive cells in specific cortical areas suggested that we should investigate the behavior of our mouse model. Sleep and circadian rhythm alterations have been observed in SZ patients, with a prevalence of up to 80% (Cohrs, 2008; Wulff et al., 2011). Such disruptions are severe and consist of advanced/delayed sleep phases or fragmented/excessive sleep periods (Wulff et al., 2011; Chan et al., 2017; Cosgrave et al., 2018). Interestingly, there are reports of a link between sleep alterations and psychotic symptoms, with increased risk of suffering one psychotic symptom when sleep disruption occurs (Lee et al., 2012; Koyanagi and Stickley, 2015; Oh et al., 2016). Moreover, sleep and circadian rhythm irregularities have also been described in genetic mouse models of SZ (Jeans et al., 2007; Yang et al., 2009; Oliver et al., 2012; Bhardwaj et al., 2015; Tam et al., 2015). We observed an alteration of the voluntary activity in the *Lis1/sLis1* mutant mice compared to the controls. The mutant animals made significantly fewer rotations than the controls. These differences were statistically significant in daytime periods, during which *Lis1/sLis1* mutant mice had longer rest periods than control mice. Other mouse models of SZ displayed a reduced amplitude of rest-activity or fragmented rhythms under constant dark conditions (Piggins, 2003; Oliver and Davies, 2009).

Our study also demonstrated that Lis*1/sLis1* mice demonstrate impaired recognition memory in the NOR task. This test evaluates the perirhinal cortex-dependent, familiarity-based recognition memory. Since PERI and DG are the most important brain regions with regard to object recognition memory (Zola-Morgan et al., 1991; Winters, 2005), these results agree with that expected according to the structural and functional alterations observed in these regions. Damaged recognition memory is one of the main cognitive symptoms of SZ patients (Danion et al., 1999; Thoma et al., 2006; Tam et al., 2015). In addition, this impairment has also been reported in SZ mouse models in both genetic (Duffy et al., 2010; Pei et al., 2014) and pharmacological (Li C. et al., 2016) models.

In summary, we observed that the *Lis1/sLis1* mutant mice presented two important behavioral alterations frequently linked to a schizophrenia-like phenotype: altered spontaneous locomotor activity during daytime periods; and impaired recognition memory.

## Conclusion

Our mouse model could represent schizophrenia in two main aspects: the pathological and the symptomatic. *LIS1* is a gene previously related to risk of developing schizophrenia in humans and it could be one of the physiopathological processes underlying schizophrenia. Moreover, we have observed a group of symptoms widely observed in other models of schizophrenia and in human patients. One of the strong points of the genetic models for neurological diseases is that they are able to accurately represent the genetic basis of human disorders (Loring et al., 1996; Lipska and Weinberger, 2000). Here, we used a genetic model in which the gene *Lis1* has a mutation similar to the mutations described in humans (Cahana et al., 2001; Reiner et al., 2002). Unfortunately, a single analysis to test the fidelity of a schizophrenia mouse model is missing. Moreover, no animal can completely reproduce the symptoms of schizophrenia as they are present in humans. In this situation animal models for schizophrenia should reproduce a group of behavioral and biological phenomena relevant to schizophrenia (Lipska and Weinberger, 2000).

Schizophrenia has an important heritable component with a complex genetic basis and a strong neurodevelopmental component (Kendler et al., 1996). Based on these facts, it is important to focus the problem from perspectives other than the pharmacological one. *Lis1/sLis* mice could be considered a genetic model affecting neurodevelopment. In our work we have shown that *Lis1/sLis1* mice exhibit histological, brain activation, and behavioral abnormalities reminiscent of schizophrenia: reduction in the number of interneurons in ACA, disbalance in cellular activation assessed by c-fos expression and the alteration of behavioral tests related to these cellular deficiencies. Our results demonstrate that *Lis1/sLis1* mutant mice exhibit an important developmentally originated alteration of the inhibitory system in cortical areas engaged in associative learning, memory, and attention. We observed that the mutation of the *Lis1* gene in early development might not only alter the number of cells positive of GAD67, CR, and PV but may also modify synapse formation decreasing VGLUT1+ terminals contacting PV+ interneurons. These alterations in the developing brain may produce a dysfunction in the activation balance of the cerebral cortex, increasing the cFOS activity, affecting the developmental process and producing long-term behavioral alterations. Specific alterations observed in DG blade activation represent a new model for understanding the functional relevance of this heterogeneous pattern.

Since the phenotype described in this animal resembles characteristics and symptoms described in SZ patients and in validated animal models for SZ, we conclude that cortical dysplasia when not severe, as is the case in *Lis1/sLis1* mutant mice, represents a pathogenetic mechanism of SZ. Moreover, alteration of the *Lis1* gene expression could be related to the risk of developing SZ.

## Data Availability Statement

The original contributions presented in the study are included in the article/Supplementary Material, further inquiries can be directed to the corresponding author/s.

## Ethics Statement

The animal study was reviewed and approved by the Ethics Committee in Animal Experimentation of the Miguel Hernadez University (2016/VSC/PEA/00190 and 2017/VSC/PEA/00211).

## Author Contributions

RG-L and AP: conceptualization, investigation, and writing – original draft preparation. AE: methodology and resources. EG-B: supervision and review. SM: conceptualization, supervision, writing – review and editing, and funding acquisition. All authors contributed to the article and approved the submitted version.

## Conflict of Interest

The authors declare that the research was conducted in the absence of any commercial or financial relationships that could be construed as a potential conflict of interest.

## Footnotes

1 http://atlas.brain-map.org/

## Supplementary Material

Supplementary Figure 1 Genotyping by PCR of *sLis1* (by using one set of primers). 0.1300 kb is the *sLis1* band. Electrophoresis gel shows the molecular weight pattern, a positive control, a negative control and a representative example of an heterozygous mouse.

## References

[B1] Abdel-Naby Sayed M. Noda Y. Mahmoud Hamdy M. Mamiya T. Nagai T. Furukawa H. (2001). Enhancement of immobility induced by repeated phencyclidine injection: association with c-Fos protein in the mouse brain. *Behav. Brain Res.* 124 71–76. 10.1016/s0166-4328(01)00235-2 11423167

[B2] Aggleton J. P. Brown M. W. (1999). Episodic memory, amnesia, and the hippocampal-anterior thalamic axis. *Behav. Brain Sci.* 22 425–444. 10.1017/s0140525x99002034 11301518

[B3] Akbarian S. Kim J. J. Potkin S. G. Hagman J. O. Tafazzoli A. Bunney W. E. (1995). Gene expression for glutamic acid decarboxylase is reduced without loss of neurons in prefrontal cortex of schizophrenics. *Arch. Gen. Psychiatry* 52 258–266. 10.1001/archpsyc.1995.03950160008002 7702443

[B4] Amaral D. G. Witter M. P. (1989). The three-dimensional organization of the hippocampal formation: a review of anatomical data. *Neuroscience* 31 571–591. 10.1016/0306-4522(89)90424-7 2687721

[B5] Andreasen N. C. Paradiso S. O’Leary D. S. (1998). “Cognitive dysmetria” as an integrative theory of schizophrenia: A dysfunction in cortical-subcortical-cerebellar circuitry? *Schizophr. Bull.* 24 203–218. 10.1093/oxfordjournals.schbul.a033321 9613621

[B6] Barkovich A. J. Kuzniecky R. I. Jackson G. D. Guerrini R. Dobyns W. B. (2005). A developmental and genetic classification for malformations of cortical development. *Neurology* 65 1873–1887. 10.1212/01.wnl.0000183747.05269.2d 16192428

[B7] Bazan N. G. (2005). Lipid signaling in neural plasticity, brain repair, and neuroprotection. *Mol. Neurobiol.* 32 89–103. 16077186 10.1385/MN:32:1:089

[B8] Benes F. M. Berretta S. (2001). GABAergic interneurons: implications for understanding schizophrenia and bipolar disorder. *Neuropsychopharmacology* 25 1–27. 10.1016/s0893-133x(01)00225-1 11377916

[B9] Bhardwaj S. K. Stojkovic K. Kiessling S. Srivastava L. K. Cermakian N. (2015). Constant light uncovers behavioral effects of a mutation in the schizophrenia risk gene Dtnbp1 in mice. *Behav. Brain Res.* 284 58–68. 10.1016/j.bbr.2015.01.048 25677649

[B10] Bitzenhofer S. H. Pöpplau J. A. Hanganu-Opatz I. (2020). Gamma activity accelerates during prefrontal development. *eLife* 9:e56795. 10.7554/eLife.56795 33206597 PMC7673781

[B11] Boyajyan A. Zakharyan R. Atshemyan S. Chavushyan A. Mkrtchyan G. (2016). Schizophrenia-associated Risk and Protective Variants of c-Fos Encoding Gene. *Recent Adv. DNA Gene Seq.* 9 51–57. 10.2174/2352092209666150223113334 25706621

[B12] Boyce S. Rupniak N. M. J. Steventon M. J. Cook G. Iversen S. D. (1991). Psychomotor activity and cognitive disruption attributable to NMDA, but not sigma, interactions in primates. *Behav. Brain Res.* 42 115–121. 10.1016/s0166-4328(05)80002-6 1647781

[B13] Bradshaw N. J. Soares D. C. Carlyle B. C. Ogawa F. Davidson-Smith H. Christie S. (2011). PKA phosphorylation of NDE1 is DISC1/PDE4 dependent and modulates its interaction with LIS1 and NDEL1. *J. Neurosci.* 31 9043–9054. 10.1523/jneurosci.5410-10.2011 21677187 PMC3610090

[B14] Briand L. A. Gritton H. Howe W. M. Young D. A. Sarter M. (2007). Modulators in concert for cognition: modulator interactions in the prefrontal cortex. *Prog. Neurobiol.* 83 69–91. 10.1016/j.pneurobio.2007.06.007 17681661 PMC2080765

[B15] Cahana A. Escamez T. Nowakowski R. S. Hayes N. L. Giacobini M. von Holst A. (2001). Targeted mutagenesis of Lis1 disrupts cortical development and LIS1 homodimerization. *Proc. Natl. Acad. Sci. U.S.A.* 98 6429–6434. 10.1073/pnas.101122598 11344260 PMC33485

[B16] Cahana A. Jin X. L. Reiner O. Wynshaw-Boris A. O’Neill C. (2003). A study of the nature of embryonic lethality inLIS1-/- Mice. *Mol. Reprod. Dev.* 66 134–142. 10.1002/mrd.10339 12950100

[B17] Calovi S. Mut-Arbona P. Tod P. Iring A. Nicke A. Mato S. (2020). P2X7 receptor-dependent layer-specific changes in neuron-microglia reactivity in the prefrontal cortex of a phencyclidine induced mouse model of Schizophrenia. *Front. Mol. Neurosci.* 13:566251. 10.3389/fnmol.2020.566251 33262687 PMC7686553

[B18] Canning K. J. Leung L. S. (1997). Lateral entorhinal, perirhinal, and amygdala-entorhinal transition projections to hippocampal CA1 and dentate gyrus in the rat: a current source density study. *Hippocampus* 7 643–655. 10.1002/(SICI)1098-1063(1997)7:6<643::AID-HIPO6>3.0.CO;2-F 9443060

[B19] Cardoso C. Leventer R. J. Dowling J. J. Ward H. L. Chung J. Petras K. S. (2002). Clinical and molecular basis of classical lissencephaly: mutations in the LIS1 gene (PAFAH1B1). *Hum. Mutat.* 19 4–15. 10.1002/humu.10028 11754098

[B20] Castañé A. Santana N. Artigas F. (2015). PCP-based mice models of schizophrenia: differential behavioral, neurochemical and cellular effects of acute and subchronic treatments. *Psychopharmacology* 232 4085–4097. 10.1007/s00213-015-3946-6 25943167

[B21] Castellani S. Adams P. M. (1981). Acute and chronic phencyclidine effects on locomotor activity, stereotypy and ataxia in rats. *Eur. J. Pharmacol.* 73 143–154. 10.1016/0014-2999(81)90086-8 7198045

[B22] Castillo-Gómez E. Pérez-Rando M. Bellés M. Gilabert-Juan J. Llorens J. V. Carceller H. (2017). Early social isolation stress and perinatal NMDA receptor antagonist treatment induce changes in the structure and neurochemistry of inhibitory neurons of the adult amygdala and prefrontal cortex. *eNeuro* 4:ENEURO.0034-17.2017. 10.1523/ENEURO.0034-17.2017 28466069 PMC5411163

[B23] Celada P. Lladó-Pelfort L. Santana N. Kargieman L. Troyano-Rodriguez E. Riga M. S. (2013). Disruption of thalamocortical activity in schizophrenia models: relevance to antipsychotic drug action. *Int. J. Neuropsychopharmacol.* 16 2145–2163. 10.1017/s1461145713000643 23809188

[B24] Chan M.-S. Chung K.-F. Yung K.-P. Yeung W.-F. (2017). Sleep in schizophrenia: a systematic review and meta-analysis of polysomnographic findings in case-control studies. *Sleep Med. Rev.* 32 69–84. 10.1016/j.smrv.2016.03.001 27061476

[B25] Chao W. Olson M. S. (1993). Platelet-activating factor: receptors and signal transduction. *Biochem. J.* 292(Pt 3), 617–629. 10.1042/bj2920617 8391253 PMC1134157

[B26] Chawla M. K. Guzowski J. F. Ramirez-Amaya V. Lipa P. Hoffman K. L. Marriott L. K. (2005). Sparse, environmentally selective expression of Arc RNA in the upper blade of the rodent fascia dentata by brief spatial experience. *Hippocampus* 15 579–586. 10.1002/hipo.20091 15920719

[B27] Cherubini E. Miles R. (2015). The CA3 region of the hippocampus: how is it? What is it for? How does it do it? *Front. Cell. Neurosci.* 9:19. 10.3389/fncel.2015.00019 25698930 PMC4318343

[B28] Chung D. W. Fish K. N. Lewis D. A. (2016). Pathological basis for deficient excitatory drive to cortical parvalbumin interneurons in Schizophrenia. *Am. J. Psychiatry* 173 1131–1139. 10.1176/appi.ajp.2016.16010025 27444795 PMC5089927

[B29] Cohrs S. (2008). Sleep disturbances in patients with Schizophrenia. *CNS Drugs* 22 939–962. 10.2165/00023210-200822110-00004 18840034

[B30] Cosgrave J. Wulff K. Gehrman P. (2018). Sleep, circadian rhythms, and schizophrenia: where we are and where we need to go. *Curr. Opin. Psychiatry* 31 176–182. 10.1097/yco.0000000000000419 29537983

[B31] Coyle J. T. Basu A. Benneyworth M. Balu D. Konopaske G. (2012). Glutamatergic synaptic dysregulation in schizophrenia: therapeutic implications. *Handb. Exp. Pharmacol.* 213 267–295. 10.1007/978-3-642-25758-2_10 PMC4817347 23027419

[B32] Danion J. M. Rizzo L. Bruant A. (1999). Functional mechanisms underlying impaired recognition memory and conscious awareness in patients with schizophrenia. *Arch. Gen. Psychiatry* 56 639–644. 10.1001/archpsyc.56.7.639 10401510

[B33] Del Pino I. García-Frigola C. Dehorter N. Brotons-Mas J. R. Alvarez-Salvado E. Martínez de Lagrán M. (2013). Erbb4 deletion from fast-spiking interneurons causes schizophrenia-like phenotypes. *Neuron* 79 1152–1168. 10.1016/j.neuron.2013.07.010 24050403

[B34] Di Cristo G. (2007). Development of cortical GABAergic circuits and its implications for neurodevelopmental disorders. *Clin. Genet.* 72 1–8. 10.1111/j.1399-0004.2007.00822.x 17594392

[B35] Dobyns W. B. Reiner O. Carrozzo R. Ledbetter D. H. (1993). Lissencephaly. A human brain malformation associated with deletion of the LIS1 gene located at chromosome 17p13. *JAMA* 270 2838–2842. 10.1001/jama.1993.03510230076039 7907669

[B36] Dragunow M. Faull R. L. M. (1990). MK-801 induces c-fos protein in thalamic and neocortical neurons of rat brain. *Neurosci. Lett.* 111 39–45. 10.1016/0304-3940(90)90341-6 2110635

[B37] Duffy L. Cappas E. Lai D. Boucher A. A. Karl T. (2010). Cognition in transmembrane domain neuregulin 1 mutant mice. *Neuroscience* 170 800–807. 10.1016/j.neuroscience.2010.07.042 20678553

[B38] Escamez T. Bahamonde O. Tabares-Seisdedos R. Vieta E. Martinez S. Echevarria D. (2012). Developmental dynamics of PAFAH1B subunits during mouse brain development. *J. Comp. Neurol.* 520 3877–3894. 10.1002/cne.23128 22522921

[B39] Fazzari P. Paternain A. V. Valiente M. Pla R. Luján R. Lloyd K. (2010). Control of cortical GABA circuitry development by Nrg1 and ErbB4 signalling. *Nature* 464 1376–1380. 10.1038/nature08928 20393464

[B40] Fisahn A. Neddens J. Yan L. Buonanno A. (2009). Neuregulin-1 modulates hippocampal gamma oscillations: implications for schizophrenia. *Cereb. Cortex* 19 612–618. 10.1093/cercor/bhn107 18632742 PMC2638818

[B41] Flames N. Long J. E. Garratt A. N. Fischer T. M. Gassmann M. Birchmeier C. (2004). Short- and long-range attraction of cortical GABAergic Interneurons by Neuregulin-1. *Neuron* 44 251–261. 10.1016/j.neuron.2004.09.028 15473965

[B42] Freedman R. (2003). Schizophrenia. *N. Engl. J. Med.* 349 1738–1749. 14585943 10.1056/NEJMra035458

[B43] Fung S. J. Fillman S. G. Webster M. J. Shannon Weickert C. (2014). Schizophrenia and bipolar disorder show both common and distinct changes in cortical interneuron markers. *Schizophr. Res.* 155 26–30. 10.1016/j.schres.2014.02.021 24674775

[B44] Fung S. J. Webster M. J. Sivagnanasundaram S. Duncan C. Elashoff M. Weickert C. S. (2010). Expression of interneuron markers in the dorsolateral prefrontal cortex of the developing human and in schizophrenia. *Am. J. Psychiatry* 167 1479–1488. 10.1176/appi.ajp.2010.09060784 21041246

[B45] Gabbott P. L. A. Warner T. A. Jays P. R. L. Salway P. Busby S. J. (2005). Prefrontal cortex in the rat: projections to subcortical autonomic, motor, and limbic centers. *J. Comp. Neurol.* 492 145–177. 10.1002/cne.20738 16196030

[B46] Gallitano A. L. Satvat E. Gil M. Marrone D. F. (2016). Distinct dendritic morphology across the blades of the rodent dentate gyrus. *Synapse* 70 277–282. 10.1002/syn.21900 26926290 PMC4879091

[B47] Garcia-Lopez R. Pombero A. Dominguez E. Geijo-Barrientos E. Martinez S. (2015). Developmental alterations of the septohippocampal cholinergic projection in a lissencephalic mouse model. *Exp. Neurol.* 271 215–227. 10.1016/j.expneurol.2015.06.014 26079645

[B48] Gilabert-Juan J. Belles M. Saez A. R. Carceller H. Zamarbide-Fores S. Moltó M. D. (2013). A “double hit” murine model for schizophrenia shows alterations in the structure and neurochemistry of the medial prefrontal cortex and the hippocampus. *Neurobiol. Dis.* 59 126–140. 10.1016/j.nbd.2013.07.008 23891727

[B49] Gilabert-Juan J. Varea E. Guirado R. Blasco-Ibáñez J. M. Crespo C. Nácher J. (2012). Alterations in the expression of PSA-NCAM and synaptic proteins in the dorsolateral prefrontal cortex of psychiatric disorder patients. *Neurosci. Lett.* 530 97–102. 10.1016/j.neulet.2012.09.032 23022470

[B50] Glausier J. R. Fish K. N. Lewis D. A. (2014). Altered parvalbumin basket cell inputs in the dorsolateral prefrontal cortex of schizophrenia subjects. *Mol. Psychiatry* 19 30–36. 10.1038/mp.2013.152 24217255 PMC3874728

[B51] Gonzalez-Burgos G. Cho R. Y. Lewis D. A. (2015). Alterations in cortical network oscillations and parvalbumin neurons in schizophrenia. *Biol. Psychiatry* 77 1031–1040. 10.1016/j.biopsych.2015.03.010 25863358 PMC4444373

[B52] Green M. F. Kern R. S. Braff D. L. Mintz J. (2000). Neurocognitive deficits and functional outcome in schizophrenia: Are we measuring the “right stuff”? *Schizophr. Bull.* 26 119–136. 10.1093/oxfordjournals.schbul.a033430 10755673

[B53] Guidotti A. Auta J. Davis J. M. Di-Giorgi-Gerevini V. Dwivedi Y. Grayson D. R. (2000). Decrease in reelin and glutamic acid decarboxylase67 (GAD67) expression in schizophrenia and bipolar disorder: a postmortem brain study. *Arch. Gen. Psychiatry* 57 1061–1069. 10.1001/archpsyc.57.11.1061 11074872

[B54] Hanahan (1986). LIS volume 17 Cover and Front matter. *Libyan Stud.* 17 f1–f5. 10.1017/s0263718900007007

[B55] Harrison P. J. Owen M. J. (2003). Genes for schizophrenia? Recent findings and their pathophysiological implications. *Lancet* 361 417–419. 10.1016/s0140-6736(03)12379-3 12573388

[B56] Harrison P. J. Weinberger D. R. (2005). Schizophrenia genes, gene expression, and neuropathology: on the matter of their convergence. *Mol. Psychiatry* 10 40–68. 10.1038/sj.mp.4001558 15263907

[B57] Hashimoto T. Arion D. Unger T. Maldonado-Avilés J. G. Morris H. M. Volk D. W. (2008). Alterations in GABA-related transcriptome in the dorsolateral prefrontal cortex of subjects with schizophrenia. *Mol. Psychiatry* 13 147–161. 10.1038/sj.mp.4002011 17471287 PMC2882638

[B58] Hashimoto T. Volk D. W. Eggan S. M. Pierri J. N. Sun Z. Sampson A. R. (2003). Altered gene expression in parvalbumin-containing GABA neurons in the prefrontal cortex of subjects with schizophrenia. *Schizophr. Res.* 60:71. 10.1016/s0920-9964(03)80597-2 PMC6740534 12867516

[B59] Hattori M. Adachi H. Tsujimoto M. Arai H. Inoue K. (1994). Miller-Dieker lissencephaly gene encodes a subunit of brain platelet-activating factor. *Nature* 370 216–218. 10.1038/370216a0 8028668

[B60] Hauser M. J. Isbrandt D. Roeper J. (2017). Disturbances of novel object exploration and recognition in a chronic ketamine mouse model of schizophrenia. *Behav. Brain Res.* 332 316–326. 10.1016/j.bbr.2017.06.013 28634108

[B61] Heckers S. Konradi C. (2002). Hippocampal neurons in schizophrenia. *J. Neural Transm.* 109 891–905. 10.1007/s007020200073 12111476 PMC4205576

[B62] Heckers S. Stone D. Walsh J. Shick J. Koul P. Benes F. M. (2002). Differential hippocampal expression of glutamic acid decarboxylase 65 and 67 messenger RNA in bipolar disorder and schizophrenia. *Arch. Gen. Psychiatry* 59 521–529. 10.1001/archpsyc.59.6.521 12044194

[B63] Heidbreder C. A. Groenewegen H. J. (2003). The medial prefrontal cortex in the rat: evidence for a dorso-ventral distinction based upon functional and anatomical characteristics. *Neurosci. Biobehav. Rev.* 27 555–579. 10.1016/j.neubiorev.2003.09.003 14599436

[B64] Herdegen T. Kovary K. Buhl A. Bravo R. Zimmermann M. Gass P. (1995). Basal expression of the inducible transcription factors c-Jun, JunB, JunD, c-Fos, FosB, and Krox-24 in the adult rat brain. *J. Comp. Neurol.* 354 39–56. 10.1002/cne.903540105 7615874

[B65] Hervig M. E. Thomsen M. S. Kalló I. Mikkelsen J. D. (2016). Acute phencyclidine administration induces c-Fos-immunoreactivity in interneurons in cortical and subcortical regions. *Neuroscience* 334 13–25. 10.1016/j.neuroscience.2016.07.028 27476436

[B66] Hoftman G. D. Lewis D. A. (2011). Postnatal developmental trajectories of neural circuits in the primate prefrontal cortex: identifying sensitive periods for vulnerability to schizophrenia. *Schizophr. Bull.* 37 493–503. 10.1093/schbul/sbr029 21505116 PMC3080694

[B67] Hoftman G. D. Volk D. W. Bazmi H. H. Li S. Sampson A. R. Lewis D. A. (2015). Altered cortical expression of GABA-related genes in schizophrenia: illness progression vs developmental disturbance. *Schizophr. Bull.* 41 180–191. 10.1093/schbul/sbt178 24361861 PMC4266281

[B68] Huang Y. Jiang H. Zheng Q. Fok A. H. K. Li X. Lau C. G. (2021). Environmental enrichment or selective activation of parvalbumin-expressing interneurons ameliorates synaptic and behavioral deficits in animal models with schizophrenia-like behaviors during adolescence. *Mol. Psychiatry.* 10.1038/s41380-020-01005-w [Epub ahead of print]. 33473150

[B69] Insel T. R. (2010). Rethinking schizophrenia. *Nature* 468 187–193. 10.1038/nature09552 21068826

[B70] Javitt D. C. Zukin S. R. (1991). Recent advances in the phencyclidine model of schizophrenia. *Am. J. Psychiatry* 148 1301–1308. 10.1176/ajp.148.10.1301 1654746

[B71] Jeans A. F. Oliver P. L. Johnson R. Capogna M. Vikman J. Molnar Z. (2007). A dominant mutation in Snap25 causes impaired vesicle trafficking, sensorimotor gating, and ataxia in the blind-drunk mouse. *Proc. Natl. Acad. Sci. U.S.A.* 104 2431–2436. 10.1073/pnas.0610222104 17283335 PMC1793901

[B72] Jentsch J. D. Roth R. H. (1999). The neuropsychopharmacology of phencyclidine: from NMDA receptor hypofunction to the dopamine hypothesis of schizophrenia. *Neuropsychopharmacology* 20 201–225. 10.1016/s0893-133x(98)00060-8 10063482

[B73] Jinno S. (2011). Topographic differences in adult neurogenesis in the mouse hippocampus: a stereology-based study using endogenous markers. *Hippocampus* 21 467–480. 10.1002/hipo.20762 20087889

[B74] Kargieman L. Santana N. Mengod G. Celada P. Artigas F. (2007). Antipsychotic drugs reverse the disruption in prefrontal cortex function produced by NMDA receptor blockade with phencyclidine. *Proc. Natl. Acad. Sci. U.S.A.* 104 14843–14848. 10.1073/pnas.0704848104 17785415 PMC1976198

[B75] Kato M. Dobyns W. B. (2003). Lissencephaly and the molecular basis of neuronal migration. *Hum. Mol. Genet.* 12 Spec No 1 R89–R96. 12668601 10.1093/hmg/ddg086

[B76] Keilhoff G. Becker A. Grecksch G. Wolf G. Bernstein H.-G. (2004). Repeated application of ketamine to rats induces changes in the hippocampal expression of parvalbumin, neuronal nitric oxide synthase and cFOS similar to those found in human schizophrenia. *Neuroscience* 126 591–598. 10.1016/j.neuroscience.2004.03.039 15183509

[B77] Kendler K. S. MacLean C. J. O’Neill F. A. Burke J. Murphy B. Duke F. (1996). Evidence for a schizophrenia vulnerability locus on chromosome 8p in the Irish Study of High-Density Schizophrenia Families. *Am. J. Psychiatry* 153 1534–1540. 10.1176/ajp.153.12.1534 8942448

[B78] Kim T.-W. Kang H.-S. Park J.-K. Lee S.-J. Baek S.-B. Kim C.-J. (2014). Voluntary wheel running ameliorates symptoms of MK-801-induced schizophrenia in mice. *Mol. Med. Rep.* 10 2924–2930. 10.3892/mmr.2014.2644 25323073

[B79] Koltai M. Hosford D. Guinot P. Esanu A. Braquet P. (1991a). PAF. A review of its effects, antagonists and possible future clinical implications (Part II). *Drugs* 42 174–204. 10.2165/00003495-199142020-00002 1717219

[B80] Koltai M. Hosford D. Guinot P. Esanu A. Braquet P. (1991b). Platelet activating factor (PAF). A review of its effects, antagonists and possible future clinical implications (Part I). *Drugs* 42 9–29. 10.2165/00003495-199142010-00002 1718687

[B81] Kontkanen O. Lakso M. Wong G. Castrén E. (2002). Chronic antipsychotic drug treatment induces long-lasting expression of fos and jun family genes and activator protein 1 complex in the rat prefrontal cortex. *Neuropsychopharmacology* 27 152–162. 10.1016/s0893-133x(02)00289-0 12093589

[B82] Kornecki E. Ehrlich Y. H. (1988). Neuroregulatory and neuropathological actions of the ether-phospholipid platelet-activating factor. *Science* 240 1792–1794. 10.1126/science.3381103 3381103

[B83] Koyanagi A. Stickley A. (2015). The association between sleep problems and psychotic symptoms in the general population: a global perspective. *Sleep* 38 1875–1885. 10.5665/sleep.5232 26085291 PMC4667394

[B84] Lee Y. J. Cho S.-J. Cho I. H. Jang J. H. Kim S. J. (2012). The relationship between psychotic-like experiences and sleep disturbances in adolescents. *Sleep Med.* 13 1021–1027. 10.1016/j.sleep.2012.06.002 22841033

[B85] Lewis D. A. Curley A. A. Glausier J. R. Volk D. W. (2012). Cortical parvalbumin interneurons and cognitive dysfunction in schizophrenia. *Trends Neurosci.* 35 57–67. 10.1016/j.tins.2011.10.004 22154068 PMC3253230

[B86] Li C. Tang Y. Yang J. Zhang X. Liu Y. Tang A. (2016). Sub-chronic Antipsychotic Drug Administration Reverses the Expression of Neuregulin 1 and ErbB4 in a Cultured MK801-Induced Mouse Primary Hippocampal Neuron or a Neurodevelopmental Schizophrenia Model. *Neurochem. Res.* 41 2049–2064. 10.1007/s11064-016-1917-x 27097547

[B87] Li J.-T. Su Y.-A. Wang H.-L. Zhao Y.-Y. Liao X.-M. Wang X.-D. (2016). Repeated Blockade of NMDA receptors during adolescence impairs reversal learning and disrupts GABAergic interneurons in rat medial prefrontal cortex. *Front. Mol. Neurosci.* 9:17. 10.3389/fnmol.2016.00017 26973457 PMC4776083

[B88] Lipska B. K. Peters T. Hyde T. M. Halim N. Horowitz C. Mitkus S. (2006). Expression of DISC1 binding partners is reduced in schizophrenia and associated with DISC1 SNPs. *Hum. Mol. Genet.* 15 1245–1258. 10.1093/hmg/ddl040 16510495

[B89] Lipska B. K. Weinberger D. R. (2000). To model a psychiatric disorder in animals: schizophrenia as a reality test. *Neuropsychopharmacology* 23 223–239. 10.1016/s0893-133x(00)00137-8 10942847

[B90] Loring J. F. Paszty C. Rose A. McIntosh T. K. Murai H. Pierce J. E. (1996). Rational design of an animal model for Alzheimer’s disease: introduction of multiple human genomic transgenes to reproduce AD pathology in a rodent. *Neurobiol. Aging* 17 173–182. 10.1016/0197-4580(95)02076-4 8744398

[B91] Lyon L. Saksida L. M. Bussey T. J. (2012). Spontaneous object recognition and its relevance to schizophrenia: a review of findings from pharmacological, genetic, lesion and developmental rodent models. *Psychopharmacology* 220 647–672. 10.1007/s00213-011-2536-5 22068459

[B92] Marín O. Rubenstein J. L. R. (2003). Cell migration in the forebrain. *Annu. Rev. Neurosci.* 26 441–483. 12626695 10.1146/annurev.neuro.26.041002.131058

[B93] Marrone D. F. Ramirez-Amaya V. Barnes C. A. (2012). Neurons generated in senescence maintain capacity for functional integration. *Hippocampus* 22 1134–1142. 10.1002/hipo.20959 21695743 PMC3367380

[B94] Moghaddam B. Adams B. Verma A. Daly D. (1997). Activation of glutamatergic neurotransmission by ketamine: a novel step in the pathway from NMDA receptor blockade to dopaminergic and cognitive disruptions associated with the prefrontal cortex. *J. Neurosci.* 17 2921–2927. 10.1523/jneurosci.17-08-02921.1997 9092613 PMC6573099

[B95] Nabeshima T. Fukaya H. Yamaguchi K. Ishikawa K. Furukawa H. Kameyama T. (1987). Development of tolerance and supersensitivity to phencyclidine in rats after repeated administration of phencyclidine. *Eur. J. Pharmacol.* 135 23–33. 10.1016/0014-2999(87)90753-9 3569423

[B96] Nakazawa K. Zsiros V. Jiang Z. Nakao K. Kolata S. Zhang S. (2012). GABAergic interneuron origin of schizophrenia pathophysiology. *Neuropharmacology* 62 1574–1583. 10.1016/j.neuropharm.2011.01.022 21277876 PMC3090452

[B97] Näkki R. Sharp F. R. Sagar S. M. Honkaniemi J. (1996). Effects of phencyclidine on immediate early gene expression in the brain. *J. Neurosci. Res.* 45 13–27. 10.1002/(sici)1097-4547(19960701)45:1<13::aid-jnr2>3.0.co;2-k 8811509

[B98] Oh H. Y. Singh F. Koyanagi A. Jameson N. Schiffman J. DeVylder J. (2016). Sleep disturbances are associated with psychotic experiences: findings from the national comorbidity survey replication. *Schizophr. Res.* 171 74–78. 10.1016/j.schres.2016.01.018 26805412

[B99] Oliver P. L. Davies K. E. (2009). Interaction between environmental and genetic factors modulates schizophrenic endophenotypes in the Snap-25 mouse mutant blind-drunk. *Hum. Mol. Genet.* 18 4576–4589. 10.1093/hmg/ddp425 19729413 PMC2773274

[B100] Oliver P. L. Sobczyk M. V. Maywood E. S. Edwards B. Lee S. Livieratos A. (2012). Disrupted circadian rhythms in a mouse model of schizophrenia. *Curr. Biol.* 22 314–319. 10.1016/j.cub.2011.12.051 22264613 PMC3356578

[B101] Ozdemir H. Ertugrul A. Basar K. Saka E. (2012). Corrigendum to “Differential Effects of Antipsychotics on Hippocampal Presynaptic Protein Expressions and Recognition Memory in a Schizophrenia Model in Mice” [Prog Neuropsychopharmacol Biol Psychiatry 39 (2012) 1–218]. *Prog. Neuropsychopharmacol. Biol. Psychiatry* 39:388. 10.1016/j.pnpbp.2012.08.006 22640753

[B102] Paus T. (2001). Primate anterior cingulate cortex: Where motor control, drive and cognition interface. *Nat. Rev. Neurosci.* 2 417–424. 10.1038/35077500 11389475

[B103] Pei J.-C. Liu C.-M. Lai W.-S. (2014). Distinct phenotypes of new transmembrane-domain neuregulin 1 mutant mice and the rescue effects of valproate on the observed schizophrenia-related cognitive deficits. *Front. Behav. Neurosci.* 8:126. 10.3389/fnbeh.2014.00126 24782733 PMC3995064

[B104] Picard N. Strick P. L. (1996). Motor areas of the medial wall: a review of their location and functional activation. *Cereb. Cortex* 6 342–353. 10.1093/cercor/6.3.342 8670662

[B105] Pietersen C. Y. Bosker F. J. Doorduin J. Jongsma M. E. Postema F. Haas J. V. (2007). An animal model of emotional blunting in schizophrenia. *PLoS One* 2:e1360. 10.1371/journal.pone.0001360 18159243 PMC2137950

[B106] Piggins H. (2003). The roles of vasoactive intestinal polypeptide in the mammalian circadian clock. *J. Endocrinol.* 177 7–15. 10.1677/joe.0.1770007 12697032

[B107] Pilz D. T. Macha M. E. Precht K. S. Smith A. C. Dobyns W. B. Ledbetter D. H. (1998). Fluorescence in situ hybridization analysis with LIS1 specific probes reveals a high deletion mutation rate in isolated lissencephaly sequence. *Genet. Med.* 1 29–33. 10.1097/00125817-199811000-00007 11261426

[B108] Post R. M. (1992). Transduction of psychosocial stress into the neurobiology of recurrent affective disorder. *Am. J. Psychiatry* 149 999–1010. 10.1176/ajp.149.8.999 1353322

[B109] Ramirez-Amaya V. Marrone D. F. Gage F. H. Worley P. F. Barnes C. A. (2006). Integration of new neurons into functional neural networks. *J. Neurosci.* 26 12237–12241. 10.1523/jneurosci.2195-06.2006 17122048 PMC6675440

[B110] Reiner O. (2000). LIS1. *Neuron* 28 633–636. 10.1016/s0896-6273(00)00142-2 11163254

[B111] Reiner O. Albrecht U. Gordon M. Chianese K. A. Wong C. Gal-Gerber O. (1995). Lissencephaly gene (LIS1) expression in the CNS suggests a role in neuronal migration. *J. Neurosci.* 15 3730–3738. 10.1523/jneurosci.15-05-03730.1995 7751941 PMC6578245

[B112] Reiner O. Cahana A. Escamez T. Martinez S. (2002). LIS1-no more no less. *Mol. Psychiatry* 7 12–16. 10.1038/sj.mp.4000975 11803439

[B113] Reynolds G. P. Abdul-Monim Z. Neill J. C. Zhang Z.-J. (2004). Calcium binding protein markers of GABA deficits in schizophrenia — post mortem studies and animal models. *Neurotox. Res.* 6 57–61. 10.1007/bf03033297 15184106

[B114] Ridderinkhof K. R. Ullsperger M. Crone E. A. Nieuwenhuis S. (2004). The role of the medial frontal cortex in cognitive control. *Science* 306 443–447. 10.1126/science.1100301 15486290

[B115] Ross C. A. Margolis R. L. Reading S. A. J. Pletnikov M. Coyle J. T. (2006). Neurobiology of schizophrenia. *Neuron* 52 139–153. 17015232 10.1016/j.neuron.2006.09.015

[B116] Rushworth M. F. S. Buckley M. J. Behrens T. E. J. Walton M. E. Bannerman D. M. (2007). Functional organization of the medial frontal cortex. *Curr. Opin. Neurobiol.* 17 220–227. 10.1016/j.conb.2007.03.001 17350820

[B117] Sagar S. M. Sharp F. R. Curran T. (1988). Expression of c-fos protein in brain: metabolic mapping at the cellular level. *Science* 240 1328–1331. 10.1126/science.3131879 3131879

[B118] Santana N. Troyano-Rodriguez E. Mengod G. Celada P. Artigas F. (2011). Activation of thalamocortical networks by the N-methyl-D-aspartate receptor antagonist phencyclidine: reversal by clozapine. *Biol. Psychiatry* 69 918–927. 10.1016/j.biopsych.2010.10.030 21251645

[B119] Sato D. Umino A. Kaneda K. Takigawa M. Nishikawa T. (1997). Developmental changes in distribution patterns of phencyclidine-induced c-Fos in rat forebrain. *Neurosci. Lett.* 239 21–24. 10.1016/s0304-3940(97)00879-3 9547162

[B120] Satvat E. Gheidi A. Voll S. Odintsova I. V. Marrone D. F. (2012). Location is everything: neurons born during fluoxetine treatment accumulate in regions that do not support spatial learning. *Neuropharmacology* 62 1627–1633. 10.1016/j.neuropharm.2011.11.025 22182782

[B121] Scharfman H. E. Sollas A. L. Smith K. L. Jackson M. B. Goodman J. H. (2002). Structural and functional asymmetry in the normal and epileptic rat dentate gyrus. *J. Comp. Neurol.* 454 424–439. 10.1002/cne.10449 12455007 PMC2519114

[B122] Schmidt B. Marrone D. F. Markus E. J. (2012). Disambiguating the similar: the dentate gyrus and pattern separation. *Behav. Brain Res.* 226 56–65. 10.1016/j.bbr.2011.08.039 21907247

[B123] Sharp F. R. Jasper P. Hall J. Noble L. Sagar S. M. (1991). MK-801 and ketamine induce heat shock protein HSP72 in injured neurons in posterior cingulate and retrosplenial cortex. *Ann. Neurol.* 30 801–809. 10.1002/ana.410300609 1838680

[B124] Stafforini D. M. McIntyre T. M. Zimmerman G. A. Prescott S. M. (2003). Platelet-activating factor, a pleiotrophic mediator of physiological and pathological processes. *Crit. Rev. Clin. Lab. Sci.* 40 643–672. 10.1080/714037693 14708958

[B125] Stansfield K. H. Ruby K. N. Soares B. D. McGlothan J. L. Liu X. Guilarte T. R. (2015). Early-life lead exposure recapitulates the selective loss of parvalbumin-positive GABAergic interneurons and subcortical dopamine system hyperactivity present in schizophrenia. *Transl. Psychiatry* 5:e522. 10.1038/tp.2014.147 25756805 PMC4354343

[B126] Stefansson H. Steinthorsdottir V. Thorgeirsson T. E. Gulcher J. R. Stefansson K. (2004). Neuregulin 1 and schizophrenia. *Ann. Med.* 36 62–71. 15000348 10.1080/07853890310017585

[B127] Sullivan E. M. O’Donnell P. (2012). Inhibitory interneurons, oxidative stress, and schizophrenia. *Schizophr. Bull.* 38 373–376. 10.1093/schbul/sbs052 22461483 PMC3329992

[B128] Tabarés-Seisdedos R. Escámez T. Martínez-Giménez J. A. Balanzá V. Salazar J. Selva G. (2006). Variations in genes regulating neuronal migration predict reduced prefrontal cognition in schizophrenia and bipolar subjects from mediterranean Spain: a preliminary study. *Neuroscience* 139 1289–1300. 10.1016/j.neuroscience.2006.01.054 16549273

[B129] Tabarés-Seisdedos R. Mata I. Escámez T. Vieta E. López-Ilundain J. M. Salazar J. (2008). Evidence for association between structural variants in lissencephaly-related genes and executive deficits in schizophrenia or bipolar patients from a Spanish isolate population. *Psychiatr. Genet.* 18 313–317. 10.1097/ypg.0b013e3283118725 19018238

[B130] Tam S. K. E. Pritchett D. Brown L. A. Foster R. G. Bannerman D. M. Peirson S. N. (2015). Sleep and circadian rhythm disruption and recognition memory in schizophrenia. *Methods Enzymol.* 552 325–349. 10.1016/bs.mie.2014.10.008 25707284

[B131] Tamamaki N. Yanagawa Y. Tomioka R. Miyazaki J.-I. Obata K. Kaneko T. (2003). Green fluorescent protein expression and colocalization with calretinin, parvalbumin, and somatostatin in the GAD67-GFP knock-in mouse. *J. Comp. Neurol.* 467 60–79. 10.1002/cne.10905 14574680

[B132] Tavares R. F. Corrêa F. M. A. (2006). Role of the medial prefrontal cortex in cardiovascular responses to acute restraint in rats. *Neuroscience* 143 231–240. 10.1016/j.neuroscience.2006.07.030 16938408

[B133] Thoma P. Zoppelt D. Wiebel B. Daum I. (2006). Recollection and familiarity in negative schizophrenia. *Neuropsychologia* 44 430–435. 10.1016/j.neuropsychologia.2005.05.017 15993449

[B134] Thomases D. R. Cass D. K. Tseng K. Y. (2013). Periadolescent exposure to the NMDA receptor antagonist MK-801 impairs the functional maturation of local GABAergic circuits in the adult prefrontal cortex. *J. Neurosci.* 33 26–34. 10.1523/jneurosci.4147-12.2013 23283319 PMC3544161

[B135] Thomsen M. S. Hansen H. H. Mikkelsen J. D. (2010). Opposite effect of phencyclidine on activity-regulated cytoskeleton-associated protein (Arc) in juvenile and adult limbic rat brain regions. *Neurochem. Int.* 56 270–275. 10.1016/j.neuint.2009.10.011 19897002

[B136] Ting A. K. Chen Y. Wen L. Yin D.-M. Shen C. Tao Y. (2011). Neuregulin 1 promotes excitatory synapse development and function in GABAergic interneurons. *J. Neurosci.* 31 15–25. 10.1523/jneurosci.2538-10.2011 21209185 PMC3078582

[B137] Valdés-Sánchez L. Escámez T. Echevarria D. Ballesta J. J. Tabarés-Seisdedos R. Reiner O. (2007). Postnatal alterations of the inhibitory synaptic responses recorded from cortical pyramidal neurons in the Lis1/sLis1 mutant mouse. *Mol. Cell. Neurosci.* 35 220–229. 10.1016/j.mcn.2007.02.017 17433713

[B138] Vertes R. P. (2006). Interactions among the medial prefrontal cortex, hippocampus and midline thalamus in emotional and cognitive processing in the rat. *Neuroscience* 142 1–20. 10.1016/j.neuroscience.2006.06.027 16887277

[B139] Vogt B. A. (2005). Pain and emotion interactions in subregions of the cingulate gyrus. *Nat. Rev. Neurosci.* 6 533–544. 10.1038/nrn1704 15995724 PMC2659949

[B140] Wakuda T. Iwata K. Iwata Y. Anitha A. Takahashi T. Yamada K. (2015). Perinatal asphyxia alters neuregulin-1 and COMT gene expression in the medial prefrontal cortex in rats. *Prog. Neuropsychopharmacol. Biol. Psychiatry* 56 149–154. 10.1016/j.pnpbp.2014.08.002 25194460

[B141] Wang Y. Baraban S. C. (2008). Aberrant dentate gyrus cytoarchitecture and fiber lamination in Lis1 mutant mice. *Hippocampus* 18 758–765. 10.1002/hipo.20434 18446829

[B142] Warburton E. C. Brown M. W. (2010). Findings from animals concerning when interactions between perirhinal cortex, hippocampus and medial prefrontal cortex are necessary for recognition memory. *Neuropsychologia* 48 2262–2272. 10.1016/j.neuropsychologia.2009.12.022 20026141

[B143] Williams N. M. Preece A. Spurlock G. Norton N. Williams H. J. Zammit S. (2003). Support for genetic variation in neuregulin 1 and susceptibility to schizophrenia. *Mol. Psychiatry* 8 485–487. 10.1038/sj.mp.4001348 12808428

[B144] Winters B. D. (2005). Glutamate receptors in perirhinal cortex mediate encoding, retrieval, and consolidation of object recognition memory. *J. Neurosci.* 25 4243–4251. 10.1523/jneurosci.0480-05.2005 15858050 PMC6725103

[B145] Woo T.-U. W. Kim A. M. Viscidi E. (2008). Disease-specific alterations in glutamatergic neurotransmission on inhibitory interneurons in the prefrontal cortex in schizophrenia. *Brain Res.* 1218 267–277. 10.1016/j.brainres.2008.03.092 18534564 PMC2665281

[B146] Wulff K. Dijk D.-J. Middleton B. Foster R. G. Joyce E. M. (2011). Sleep and circadian rhythm disruption in schizophrenia. *Br. J. Psychiatry* 200 308–316. 10.1192/bjp.bp.111.096321 22194182 PMC3317037

[B147] Wynshaw-Boris A. (2007). Lissencephaly and LIS1: insights into the molecular mechanisms of neuronal migration and development. *Clin. Genet.* 72 296–304. 10.1111/j.1399-0004.2007.00888.x 17850624

[B148] Yang K. Trepanier C. H. Li H. Beazely M. A. Lerner E. A. Jackson M. F. (2009). Vasoactive intestinal peptide acts via multiple signal pathways to regulate hippocampal NMDA receptors and synaptic transmission. *Hippocampus* 19 779–789. 10.1002/hipo.20559 19173226 PMC2736340

[B149] Zhang Z. J. Reynolds G. P. (2002). A selective decrease in the relative density of parvalbumin-immunoreactive neurons in the hippocampus in schizophrenia. *Schizophr. Res.* 55 1–10. 10.1016/s0920-9964(01)00188-8 11955958

[B150] Zola-Morgan S. Squire L. R. Alvarez-Royo P. Clower R. P. (1991). Independence of memory functions and emotional behavior: separate contributions of the hippocampal formation and the amygdala. *Hippocampus* 1 207–220. 10.1002/hipo.450010208 1669294

