# Supplementary figures and images for: Interneuron Heterotopia in the Lis1 Mutant Mouse Cortex Underlies a Structural and Functional Schizophrenia-Like Phenotype
Source: Front Cell Dev Biol. 2021 Jul 13;9:693919. doi: 10.3389/fcell.2021.693919 (PMC8313859; doi:10.3389/fcell.2021.693919)

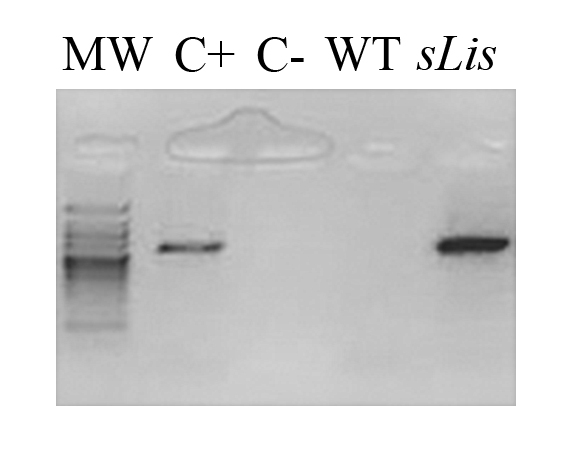

Supplement: Supplementary Figure 1 — Genotyping by PCR of sLis1 (by using one set of primers). 0.1300 kb is the sLis1 band. Electrophoresis gel shows the molecular weight pattern, a positive control, a negative control and a representative example of an heterozygous mouse. [file Image_1.JPEG]
